# Criminal recidivism rates globally: A 6-year systematic review update

**DOI:** 10.1016/j.jcrimjus.2023.102115

**Published:** 2023

**Authors:** Denis Yukhnenko, Leen Farouki, Seena Fazel

**Affiliations:** Department of Psychiatry, University of Oxford, Warneford Lane, OX3 7JX Oxford, UK.

**Keywords:** Recidivism, Reoffending, Re-entry, Prison, Community sentence, Probation

## Abstract

**Objectives:**

Previous work has shown that direct comparison of recidivism rates between jurisdictions without accounting for potential sources of their variation can be misleading. We aimed to systemically review data on recidivism rate internationally and explore sources of between-country variation.

**Methods:**

We reviewed recidivism rates in individuals released from prison and given community sentences. We systematically searched peer-reviewed and gray literature focusing on publications since a systematic review in mid-2019. We extracted data on reoffending, reimprisonment, and re-arrests. To examine the association between index offences and recidivism rates, we calculated risk ratios. We used meta-regression to estimate the association between recidivism in released prisoners and country-level variables. We also summarised reported effects of the COVID-19 pandemic on recidivism rates.

**Results:**

Recidivism data were found for 33 countries. Released prisoners had 2-year reconviction rates between 18% and 55%, while individuals given community sentences had rates between 10% and 47%. Recidivism rates varied based on proportions of index offences. Country-level factors like homicide, robbery, and imprisonment rates were associated with prisoner recidivism. Lower rates during COVID-19 were linked to disruptions in criminal justice processes, reduced prison populations, and fewer crime opportunities.

**Conclusions:**

Interpreting recidivism rates requires considering individual and country-level factors. Transparent reporting of these factors is needed.

## 1 Introduction

Criminal recidivism rates are often used as a key indicator of the effectiveness of criminal justice systems. In particular, they are used to decide what offender management programmes are rolled out widely (e.g., Robinson et al., 2021). Recidivism rates are routinely collected by most countries and reported by specific governmental agencies (see Federal Statistical Office, 2015; Ministry of Justice, 2023; SPAC, 2018). Recidivism is measured in different ways, and previous reviews have shown that the 2-year reconviction rate is most commonly reported outcome in individuals released from prisons (Yukhnenko, Sridhar, & Fazel, 2019) and those completing community sentences (Yukhnenko, Wolf, Blackwood, & Fazel, 2019).

Recidivism rates are primarily used to assess the performance of national and state justice systems or rehabilitation programmes over time. Governments and agencies often strive to implement policy and practice changes that lead to lower recidivism rates. However, reported recidivism rates are determined by many factors, many of which are not necessarily associated with the effectiveness of the rehabilitation and reintegration of sentenced individuals. These include the source of data, the definition of recidivism used, and the follow-up period (Andersen & Skardhamar, 2017; Yukhnenko, Sridhar, & Fazel, 2019). Recidivism rates should also be interpreted in the context of general crime rates that depend heavily on general economic and demographic factors (Anser et al., 2020). Given the complex, multicausal nature of reported recidivism rates, their use for comparison between countries and jurisdictions is often problematic. However, despite this, recidivism rates are frequently and inappropriately employed to compare the effectiveness of different approaches to criminal justice between different countries, especially by the media (BBC News, 2019; Browne, 2020). Such comparisons pose challenges due to the varying ways recidivism is operationalized, measured, and reported across different jurisdictions. Comparing recidivism rates between different countries is more likely to reflect variations in reporting practices or in other related factors than the meaningful differences in effectiveness of rehabilitation and reintegration programs for individuals released from prison or given community sentences. Understanding the factors that explain heterogeneity in between-country recidivism rates is important for policymakers, researchers and practitioners to ensure the effects of policy changes and practices are evaluated correctly. Moreover, understanding the factors contributing to reported recidivism rates across jurisdictions could assist in revising and improving how recidivism is reported, ensuring that it better captures outcomes of criminal justice agencies.

One of the factors contributing to recidivism rates that has not received adequate attention in the literature is the proportion of individuals with different index offences among the cohort of individuals released from prison or given community sentences. For instance, if most individuals released in a particular year have been sentenced for a highly recurrent offence (e.g., property crime), it would lead to an overall increase in the recidivism rate for the studiedcohort compared to a cohort where less people were sentenced for a property crime. Other potential contributors to reported recidivism rates include macroeconomic and general criminological factors within a country. Previous research has explored the relationship between crime rates, poverty, inequality, and economic growth (Anser et al., 2020; Gruszczyńska & Gruszczyński, 2023; Wolf, Gray, & Fazel, 2014). However, the connection between economic factors, specific crime rates, and recidivism rates remains unexamined. All of these factors exhibit significant variations between different countries and can also vary within the same country, which can dilute any direct effects of the criminal justice system on reported recidivism rate.

In the present study, we systematically reviewed studies on recidivism rates in individuals released from prison and those given community sentences. The primary aim was to provide a current overview of recidivism information and reporting practices. Our secondary aim was to examine possible explanations for the expected variations in the reported between-country recidivism rates. Potential explanatory factors include criminological variables in the analysed cohorts, such as differences in the proportions of index crimes, as well as country-level variables such as GDP per capita, incarceration rate, murder rate, robbery rate, and inequality as measured by Gini coefficient. Additionally, we summarized the effects of COVID-19 pandemic on reported recidivism rates.

## 2 Methods

This review is an update of three systematic reviews (Fazel & Wolf, 2015; Yukhnenko, Sridhar, & Fazel, 2019; Yukhnenko, Wolf, et al., 2019). We searched SAGE, MEDLINE, EMBASE, PsycINFO, PsycARTICLES, and Web of Science bibliometric databases using search terms related to criminal recidivism. The keywords included 50 countries with largest prison populations in absolute terms in 2022 (World Prison Brief, 2022) and a list of commonly reported outcomes (see Appendix A for exact search terms). Those would be the countries where successful interventions would have the greatest population impact.

We used Google Scholar and Google Web for subsequent targeted searches of gray literature. In addition, we scanned reference lists of included documents. If titles and abstract contained relevant keywords and did not contain any terms matching exclusion criteria, then the full-text publication was screened for appropriate data. If multiple reports were identified for the same country, we extracted the most recent data for a given outcome. If no new data for a given country were identified, we included studies from the previous reviews (Yukhnenko, Sridhar, & Fazel, 2019; Yukhnenko, Wolf, et al., 2019).

Studies for geographical regions within a country were included if national information was unavailable or dated. We followed the PRISMA guidelines (see the checklist in Supplementary material), and a corresponding flow chart is provided in Appendix B.

We included cohorts where data on reconviction, re-arrest, and re-imprisonment rates in released prisoners and/or individuals given community sentences were reported. We excluded studies that focused on recidivism in selected populations (such as young offenders or sex offenders) and intervention studies. We also excluded studies where the outcome definition was unclear or not reported. We extracted recidivism data separately for released prisoners and individuals given community sentences.

LF and DY conducted the search and independently extracted the data on country, sample selection, definitions of outcomes and rates. Included studies were accessed using the NIH Quality Assessment Tool for Before-After (Pre-Post) Studies With No Control Group (NIH, 2021). Uncertainties were checked with SF. Publications in languages other than English were translated.

To examine the association between different index offences and recidivism, we extracted relevant data from studies that reported recidivism rates for different groups of index offences and provided corresponding cohort sizes. As such data were only available for mixed cohorts of individuals (i.e., combining released prisoners and community sentences), we did not separate this analysis by sentence type. We extracted recidivism rates in individuals sentenced for the most commonly reported offence categories: violent, sexual, property, drugs, and traffic. For all included cohorts, if an individual was sentenced for several crimes, the most serious index crime was used. Prior criminal history was not taken into account. As the violent crime category had the most consistent definition, we used it as the reference category to calculate risk ratios (RRs) for other types of index offences. We pooled the RRs within the same index offence category by applying the Mantel-Haenszel method for random-effects estimation using the meta package for R (Balduzzi, Rücker, & Schwarzer, 2019).

To explore potential sources of variation in reported recidivism rates in prisoners, for a given year of their release, we extracted country-level variables for studies that reported 2-year reconviction rate, which was the most reported recidivism outcome. We additionally searched governmental reports and international agencies' website for the corresponding information. The extracted variables included incarceration rates, murder rate, robbery rate, Gini index, and GDP per capita. Our analyses were limited to countries where 2-year reconviction data, cohort sizes, and corresponding country-level variables were available. The 2-year reconviction rate was chosen as it was the most commonly reported outcome. These were based on previous studies that have shown ecological associations with crime and their reliable reporting for most countries (Wolf et al., 2014). The variables can be interpreted as proxies for the general socioeconomic situation and criminal justice in a particular country. To estimate the association of the country-level variables with the reported recidivism rate, we fitted a series of univariate meta-regression models using the weighted least squares method. The regression was implemented with statsmodels library for Python (Seabold & Perktold, 2010). To additionally examine the association between crime rates and recidivism within a single region, we extracted the rates of general, violent, and property crime for Nordic countries for the same reporting year.

## 3 Results

We identified 37 new publications from 33 countries and territories reporting on recidivism among released prisoners and those given community sentences that met the specified inclusion criteria. We identified 5 additional publications from Germany, Italy, Iceland, Norway, and Sweden compared with the previous review (Yukhnenko, Wolf, et al., 2019). Ten of the 50 countries with the largest prison populations had recidivism data meeting inclusion criteria (Argentina, Australia, Brazil, Canada, Chile, England and Wales, France, Germany, Italy, and South Korea). All newly identified data were published by governmental agencies, except for one source (Morgan and Morgan, 2019). The extracted data, outcome definition, and other relevant information for each individual country are presented in supplementary Vignettes. In addition, during screening, 23 studies that reported recidivism data using cross-sectional methods were identified. These did not meet inclusion criteria and excluded from the analysis (see references to these sources in Appendix C).

Recidivism data for people released from prison were available for 33 countries. A two-year reconviction was the most commonly reported outcome. In released prisoners, 2-year reconviction rates ranged from 17.6% in Norway to 54.9% in Australia (Table 1). For community-sentenced individuals, data were available for 20 countries, with a two-year reconviction being most commonly reported. The 2-year reconviction rates for community sentenced individuals ranged from 9.7% in Chile to 46.6% in Denmark (Table 2). The reimprisonments rates are presented in Appendix D.

**Table 1:** Reconviction rates in individuals released from prison

			Length of the follow-up period (years)					
Country	Year of release	Cohort size	1	2	3	4	5	Publication
*Europe*								
Nordic countries								
Denmark*	2018	2710		32.0				Kristoffersen (2022)
Finland*	2018	2776		33.0				Kristoffersen (2022)
Iceland*	2018	151		21.2				Kristoffersen (2022)
Norway*	2018	4509		17.6				Kristoffersen (2022)
Sweden*	2018	7959		32.0				Kristoffersen (2022)
Sweden*	2019	NA	43.0					National Council for Crime Prevention (2022)
*The United Kingdom*								
England and Wales	2020	48,843	38.9					Ministry of Justice (2022)
Northern Ireland	2018–2019	1309	44.9					Department of Justice (2021)
Scotland**	2018–2019	5549	43.8					Scottish Government (2021)
*Other*								
Austria	2017	6607	13.5	24.3	30.3	34.7		Statistics Austria (2023)
Estonia	2015–2017	NA		32.0				Ahven et al. (2019)
Ireland, Republic of	2019	4026	44.6					Central Statistics Office Ireland (2022a)
Ireland, Republic of	2016	2626			62.3			Central Statistics Office Ireland (2022a)
Germany	2007	26,602				46.0		Jehle (2014)
France	2016	NA	32.9	45.4				Ministère de la Justice (2022)
Latvia	2009	NA		51.0				Kipena, Zavackis, & Nikisins (2012)
Netherlands	2017	23,302	18.8	24.6				Ministry of Justice (2017)
Switzerland	2016	1393			44.7			Federal Statistical Office (2018)
Poland	2012	30,899	16.6	28.3	35.9	39.9	40.4	Jaki (2018)

*Asia*								
Malaysia	2017	NA	9.0					Wahab (2019)
Taiwan	2014	NA	27.4	42.7	51,3	56.6		Tsai and Wu (2022)
Taiwan	2015	NA	28.7	41.2	52.4			Tsai and Wu (2022)
Taiwan	2016	NA	30.9	52.0				Tsai and Wu (2022)
Taiwan	2017	NA	31.0					Tsai and Wu (2022)
Singapore	2019	10,570		19.1				SPS (2021)

*Oceania*								
Australia	2019–2020	NA		54.9				Australian Government (2021)
New Zealand	2020–2021	NA	36.0					Department of Corrections (2022)

*South America*								
Chile	2011	20,867		39.1				Gendarmería de Chile (2016)

*North America*								
Canada (federal)	2011–2012	8893	17.1	27.9	35.4		43.2	Stewart, Wilton, Baglole, & Miller (2019)
Canada (Ontario)	2015–2016	NA		37.0				Government of Ontario (2021)
USA (33 states)	2012	408,300	36.8	52.9	61.5	67.0	70.8	U.S. Department of Justice (2021)

The follow-up period for Latvia is 29 months. Data reported for cohorts aged 18 and older unless indicated otherwise. *Reported for cohorts aged 15 and older.

**Table 2:** Reconviction rates in community sentenced individuals

		Length of the follow-up period (years)						
Country	Year of release	Cohort size	1	2	3	4	5	Publication
*Europe*								
Nordic countries								
Denmark*	2018	7387		45.6				Statistics Denmark (2023)
Finland*	2005	3767		25.6				Graunbøl et al. (2010)
Iceland*	2005	73		16.4				Graunbøl et al. (2010)
Norway*	2005	2839		19.8				Graunbøl et al. (2010)
Sweden*	2008	22,306	23.8	32.8	38.1			National Council for Crime Prevention (2017)
*The United Kingdom*								
England and Wales	2020	50,136	28.0					Ministry of Justice (2022)
Northern Ireland	2018–2019	3308	21.2					Department of Justice (2021)
Scotland**	2018–2019	27,210	25.2					Scottish Government (2021)
*Other*								
Austria	2017	10,636	11.2	21.3	27.3	31.6		Statistics Austria (2023)
Czech Republic*	2012	4233		48.1				Tomášek and Rozum (2018)
France	2004	241,999	9.1	18.1	25.2	34.2		Ministère de la Justice (2013)
Ireland, Republic of	2018	4999	28.0					Central Statistics Office Ireland (2022b)
Ireland, Republic of	2017	4909	29.0	41.0				Central Statistics Office Ireland (2022b)
Ireland, Republic of	2016	4447	31.0	43.0	48.0			Central Statistics Office Ireland (2022b)
Germany	2007	96,521				39.0		Jehle (2014)
Latvia	2009	1190		17.0				Kipena et al. (2012)
Netherlands	2017	36,095	38.8	30.3				Ministry of Justice (2017)

*Oceania*								
Australia	2019–2020	NA		16.1				Australian Government (2021)
New Zealand	2020–2021	NA	19.0					Department of Corrections (2022)

*South America*								
Chile	2011	36,895		9.7				Gendarmería de Chile (2016)
Brazil	2015	NA				23.9		Conselho Nacional De Justica Brazil (2020)

*North America*								
Canada (Ontario)	2015–2016	NA		23.0				Government of Ontario (2021)

The follow-up period for Latvia is 29 months. Data reported for cohorts aged 18 and older unless indicated otherwise. *Reported for cohorts aged 15 and older. ** Reported for cohorts aged 21 and older.

**Fig. 1:**
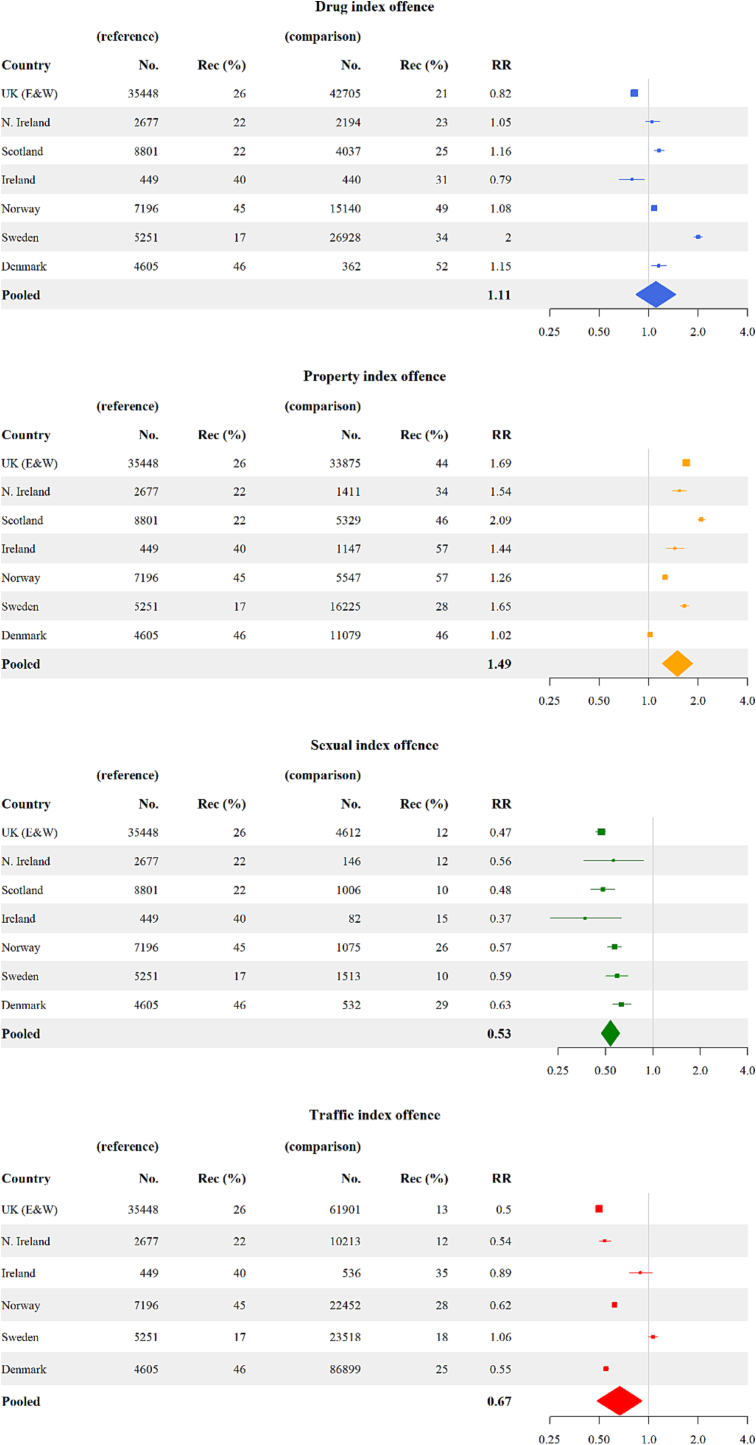
Recidivism risk in individuals sentenced for different index offences compared to individuals sentenced for violent crimes. Data were from combined cohorts of individuals (both released prisoners and community sentenced individuals). RR = relative risk; rec = proportion who recidivated.

We identified seven studies that provided recidivism data in individuals stratified by index offence (Fig. 1). None of these reported index offence data separately by prison and community sentences. Compared to individuals sentenced for violent offences, individuals sentenced for property offences had a higher recidivism rate (pooled RR = 1.49, 95% CI: 1.21–1.85). Individuals sentenced for a drug offence had comparable recidivism rates with those sentenced for violence with variations between countries (pooled RR = 1.11, 95% CI: 0.83–1.47). Individuals sentenced for sexual and traffic offences had lower recidivism rates compared to those sentenced for violent offences (sexual offences: pooled RR = 0.53, 95% CI: 0.47–0.61; traffic offences: pooled RR = 0.67, 95% CI: 0.48–0.92).

**Fig. 2:**
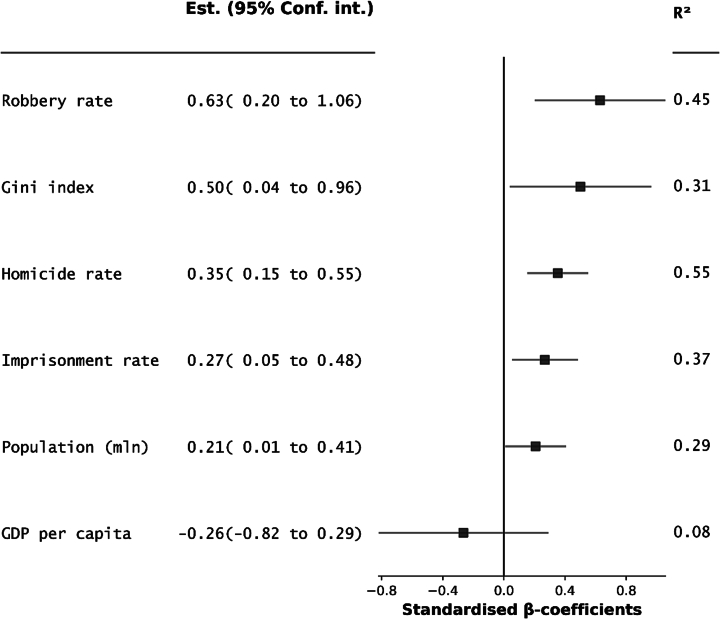
The univariate association between country-level variables and 2-year reconviction rate. The country-variables were extracted for 11 countries for a year of recidivism data reporting or, if data were unavailable, for the closest year: Austria (year of reporting: 2017), Denmark (2018), Sweden (2018), Finland (2018), Iceland (2018), Norway (2018), Netherlands (2017), Poland (2012), Singapore (2019), Chile (2011), Canada (2011), USA (2012). The standardised beta-coefficients were estimated with univariate weighted least squares regression, where weights were inverse variance of 2-year reconviction rates. R^2^ = coefficient of determination.

For 11 countries, for which country-level data on the 2-year reconviction rates and cohort sizes were available, we extracted country-level variables, reflecting economic output, income inequality, and reported crime rates (Table 3). The results of the univariate meta-regressions showed that the 2-year reconviction rate had a significant positive association with the homicide rate, robbery rate, and imprisonment rate (Fig. 2, Appendix E). The Gini index showed a weak association with the 2-year reconviction rate, which was not statistically significant. Two-year reconviction rates were not associated with GDP per capita and population size. For four Nordic countries (Denmark, Finland, Sweden, and Sweden), for 2018 reporting year, we extracted the information on country-level criminal rates (Table 4). Scandinavian countries and Finland had comparable homicide rates; however, their property and drug crime rates varied substantially.

**Table 3:** Country-level variables for meta-regression extracted for countries that reported 2-year reconviction rates in released prisoners

Country	Year	Cohort size	2-year reconviction (%)	Imprisonment per 100,000	Homicide per 100,000	GDP per capita (USD)	Gini index	Robbery per 100,000	Population (mlllion)
Austria	2017	6607	24.3	98	0.7	47,429	27.2	24.1	8.8
Denmark	2018	4909	32.0	65	0.8	61,592	28.2	31.6	5.8
Finland	2018	2776	33.0	53	1.2	49,988	27.3	25.3	5.5
Iceland	2018	151	21.2	37	0.9	74,461	26.1	14.0	0.3
Norway	2018	4509	17.6	65	1.2	82,268	27.6	58.0	5.3
Netherlands	2017	23,302	24.6	59	0.8	48,675	28.5	47.0	17.1
Poland	2012	30,899	28.3	221	1.1	13,011	33.5	43.0	37.8
Singapore	2019	10,570	19.1	199	0.2	65,831	45.2	0.9	5.7
Chile	2011	20,867	39.1	329	3.7	14,629	46.0	535.0	17.2
Canada	2011	8893	27.9	117	1.8	52,224	33.6	86.0	38.3
USA	2012	408,300	32.1	707	4.7	51,784	40.9	113.0	331.9
Sweden	2018	7959	32.0	64	1.1	54,589	30.0	82.7	10.2

Homicide includes murder (intentional homicide) and manslaughter (unintentional homicide). Sources for extracted indices per country are available in Vignettes.

**Table 4:** Country-level general and specific crime rates and 2-year reconviction rates in released prisoners in Scandinavian countries and Finland in 2018

Country	2-year reconviction rate (%)	Crime per 100,000	Homicide per 100,000	Property crime per 100,000	Drug and alcohol crime per 100,000	Population (mlllion)
Finland	32.0	8041	1.6	3752.0	872.8	5.52
Norway	17.6	6004	1.2	1753.6	863.0	5.30
Sweden	33.0	15,157	1.1	4350.3	1041.2	102.3
Denmark	32.0	8213	1.01	5295.0	299.0	5.79

Reconviction rates are reported according to University College of Norwegian Correctional Service (Kristoffersen, 2022). Homicide includes murder (intentional homicide) and manslaughter (unintentional homicide). Drug and alcohol related crime include drug trafficking, smuggling, selling, illegal possession, and driving under influence. Sources for extracted indices per country are available in Vignettes.

We additionally identified reports from nine countries that explored the impact of the COVID-19 pandemic and related restrictions on reported recidivism rates. All identified reports noted a decrease in recidivism during the pandemic likely caused by delay in court processing time and decreased opportunities to commit crime during lockdown.

Most identified studies were of good or fair quality as measured by the NIH Quality Assessment Tool (Appendix F). The most common problem with included studies was the absence of reported cohort sizes, which makes it not possible to reliably pool data.

## 4 Discussion

This systematic review synthesises criminal recidivism rates in individuals released from prison and those given community sentences from 36 studies based on around 1.4 million individuals. Only 10 out of 50 countries with the largest prison populations reported recidivism statistics. We found that 2-year reconviction was most commonly reported outcome for both populations. We examined the association between recidivism rates and different country-level determinants including the proportions with different index offences, and markers of economic output, income inequality, and general crime rates. This updated review has four main findings.

First, reported recidivism rates are generally high across most countries, with at least one in five individuals reoffending within two years. In some countries, recidivism rates exceed 40% after one year. This presents a significant societal burden in terms of public safety, healthcare, and associated costs. Recidivists are estimated to be responsible for a considerable proportion of all offences committed in any given year. In the US, repeat offending contributed to 20% of all offences (Petersilia, 2011).

Second, we identified new potential sources of variation between recidivism rates in sentenced prisoners. Previous research indicated that reported recidivism rates are sensitive to several measurement variables, including definitions, length and type of the follow-up (Andersen & Skardhamar, 2017; Yukhnenko, Sridhar, & Fazel, 2019). We found that property offences were consistently associated with the largest relative recidivism risk. Therefore, reported and detected property crimes could account for a significant portion of reported recidivism rates and a large proportion of property offenders in any cohort would increase recidivism rates.

Our results further suggest that higher imprisonment rates, robbery rates, and homicide rates within a country were associated with higher reported reconviction rates in people released from prison. Imprisonment rates may reflect the overall level of a country's criminalisation, which could increase rates of repeated crime in released individuals. Serious violent crime rates, especially homicide rates, could be used as indicators of general level of crime within a country as they have high levels of reporting and clearance (Lehti et al., 2019). In other words, the more criminogenic a society is, the higher the recidivism rates (given other factors are held constant).

The substantial contribution of property crime to overall recidivism rates may partially explain the low reported recidivism rates in Norway. All four Scandinavian countries have similar levels of serious violent crime, as indicated by similar homicide rates. We can hypothesise that the low recidivism rates in Norway, when compared to neighbouring countries, are at least partially due to low reported non-violent crime rates. This may be attributed to either true low property-related criminality, decreased reporting and detectability, or lower rates of investigation and prosecution of low-level crime.

Third, while more countries have started reporting recidivism rates in recent years, it is remains problematic to draw conclusions from them about a prison and probation system' effectiveness at rehabilitation. However, some countries, such as England and Wales, Republic of Ireland, Austria, and Australia, regularly provide detailed statistical reports on various aspects of their legal and prison systems that allow for evaluating different factors contributing to recidivism rates. These factors include reported and investigated crime, number of arrests, charges brought, court load, index sentence data, processing times, and data collection and reporting practices. This approach should be extended to other countries.

Fourth, during the COVID-19 pandemic, recidivism rates initially declined in most countries, but rebounded after the lifting of restrictions. Several factors contributed to the declines, including pandemic-related delays in reporting, processing, and data collection by reporting agencies, police, and courts. Jurisdictions also sought to reduce prison populations by deferring sentences, imposing non-custodial sentences, or reducing sentences for low-risk prisoners. Lockdowns and social distancing also limited opportunities for committing certain types of crimes, resulting in a direct reduction in recidivism events.

Overall, these findings highlight the methodological difficulties associated with recidivism reporting and comparative analysis. Even within one jurisdiction, recidivism rates are sensitive to many systemic factors associated with the police, court system, and reporting agencies. Accounting for such factors between jurisdictions requires careful and detailed analysis that needs to consider more than recidivism rates. Reporting agencies could aid in such analysis by routinely providing detailed reports using best practices (see Yukhnenko et al. (2019) for recommendations) and by creating flexible open data tools.

### 4.1 Strengths and limitations

This is the first study to systematically review recidivism rates in general populations of both released prisoners and individuals given community sentences. Studies included in the review were generally of high quality and were conducted using large samples. A novel aspect was investigating economic output, inequality, incarceration rates, and general crime rates as potential sources of variation for reconviction in released prisoners using between country comparisons. We were also able to quantitatively examine the relative risk associated with different types of index offences in the cohorts of sentenced individuals.

The substantial heterogeneity of the cohorts and outcome definitions did not allow for direct quantitative comparison of recidivism rates. Furthermore, the community sentenced cohorts differed from each other with regards to the nature of the supervision involved. For example, community supervision with mandated treatment is implemented in some countries. The estimated association between recidivism and country-level variables should also be interpreted with caution due to limited data availability for many countries. Consequently, findings may not necessarily apply to other jurisdictions. In addition, recidivism rates in those given community sentences should not be directly compared with released prisoners even within the same jurisdiction due to substantial differences between these two groups, such as proportion of index crimes within the cohorts and other background factors.

## 5 Conclusion

Recidivism rates need to be interpreted within the broader context of factors related to legal and criminal justice systems. Governmental agencies reporting these data must make efforts to regularly provide detailed and transparent background criminological data, facilitating independent analysis and pooling of results. The use of recidivism rates for international comparisons should be avoided until sufficient analysis of the underlying factors contributing to reported rates has been conducted.

## Funding

This study was supported by 10.13039/100010269Wellcome Trust [grant #202836/Z/16/Z to SF].

## Declaration of Competing Interest

None.

## Figures and Tables

**Figure:**
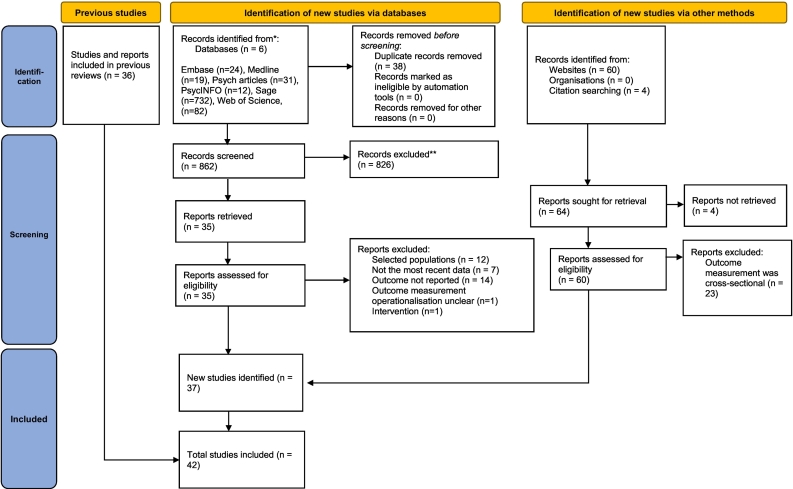


**Table:** 

Country	Prevalence (%)	Reporting period	Publication	Page with data
Reporting method does not fit inclusion criteria				
Bahamas	46.0	2016–2019	Bergman, Seepersad, Safranoff, & Cafferata (2020)	p.122
Barbados	60.0	2016–2019	Bergman et al. (2020)	p.122
Fiji	2.5	2018–2019	Fiji Corrections Service (2021)	p. 11
Guyana	34.0	2016–2019	Bergman et al. (2020)	p.122
Suriname	46.0	2016–2019	Bergman et al. (2020)	p.122
Trinidad and Tobago	49.0	2016–2019	Bergman et al. (2020)	p.122
Mexico	25.9	2016	Instituto Nacional de Estadística y Geografía (2016)	p.17
Colombia	20.4	2021	Corporación Excelencia en la Justicia (2023)	web page
Paraguay	40.0	2019	Mecanismo Nacional de Prevención de la Tortura (2019)	web page
Argentina	24.0	2020	Ministerio de Justicia y Derechos Humanos (2020)	p.41
Romania	37.3	2021	Ministerul Justitiei (2021)	p.5
Sri Lanka	22.6	2020	Statistics Division Prison Headquarters (2021)	p.45
Ghana	22.2	2011	Antwi (2015)	p.106
Brunei	53.0	N/A	Mundia, Matzin, Mahalle, Hamid, and Osman (2016)	p. 746
Indonesia	1.0	2006	Thailand Institute of Justice (2016)	p. 3–31
India	4.7	2020	National Crime Records Ministry of Home Affairs, 2020	p.130
Papua New Guinea	11.0	2019	Morgan and Morgan (2019)	p.23
No definition provided				
Albania	24.0	2019	United Nations Office on Drugs and Crime (2022)	p.16
Czechia	66.0	2019	United Nations Office on Drugs and Crime (2022)	p.16
Thailand	24.0	2016	United Nations Office on Drugs and Crime (2022)	p. 16
Unable to locate original source				
USA (nationally)	66.7	N/A	Antwi (2015)	p.3
South Africa	47.0	N/A	Antwi (2015)	p.3
Malta	17.0	N/A	Camilleri (2016)	n/a

**Table:** 

		Length of the follow-up period (years)						
Country	Year of release	Cohort size	1	2	3	4	5	
*Europe*								
Other								
Austria	2017	3819					46.6	Statistics Austria (2023)
Belgium	2003–2005	1175					62.3	Institut National de Criminalistique (2012)

*Asia*								
Israel	2014	6897	10.4	19.0	26.7	33.1	39.2	Vaknin & Ben-Zvi (2021)
Israel	2015	6766	10.6	19.4	27.1	34.3		Vaknin & Ben-Zvi (2021)
Israel	2016	6549	10.4	19.6	27.9			Vaknin & Ben-Zvi (2021)
Israel	2017	6075	12.2	22.5				Vaknin & Ben-Zvi (2021)
Israel	2019	6329	12.7					Vaknin & Ben-Zvi (2021)
Japan	2017	NA			9.0			Statistics Bureau of Japan (2017)
Thailand	2016	NA			35.4			Thailand Institute of Justice (2021)
Philippines*	2020	84,133		17.5				(Bureau of Jail Management and Penology, personal communication, October 20, 2022)
South Korea	2017	30,702			24.6			Korean Government (2022)
Switzerland	2016	1393			26.1			Federal Statistical Office (2018)

*Oceania*								
Australia	2019–2020	NA		46.0				Australian Government (2021)
New Zealand	2020–2021	NA	22.0					Department of Corrections (2022)

*South America*								
Brazil	2015	NA				42.5		Conselho Nacional De Justica Brazil (2020)

*North America*								
Jamaica	2014	NA		30.0				Department of Correctional Services (2017)


Reimprisonment rates in individuals given community sentences								
		Length of the follow-up period (years)						
Country	Year of release	Cohort size	1	2	3	4	5	Publication
*Europe*								
Other								
Austria	2017	6652					26.2	Statistics Austria (2023)

All data reported for cohorts aged 18 and older. *The outcome for 1 year and 9 months.

**Table:** 

Country-level variable	β-coefficient	Standard error	Intercept	R^2^	t-value	*p*-value
Imprisonments per 100,000 individuals	0.267439	0.109978	−0.038063	0.371598	2.431744	0.035345
Homicides per 100,000 individuals	0.351869	0.101627	−0.099839	0.545206	3.462368	0.006099
Robberies per 100,000 individuals	0.628735	0.217752	0.441990	0.454654	2.887383	0.016180
Gross Domestic Product per capita (in USD)	−0.264347	0.282821	0.519535	0.080344	−0.934681	0.371973
Population (millions)	0.206573	0.101737	0.055140	0.291924	2.030463	0.069751
Gini index	0.500521	0.236522	0.089120	0.309306	2.116174	0.060411

The regression was performed on the panel of 11 reports from 11 countries. The weighed least squares method was used for meta-regression with inverse variance as weights.

**Table:** 

Criterion	Country						
	Australia	Austria	Belgium	Brazil	Canada	Czech Republic	Chile
1. Was the study question or objective clearly stated?	✓	✓	✓	✓	✓	✓	✓
2. Were eligibility/selection criteria for the study population prespecified and clearly described?	✓	✓	✓	✓	✓	✓	✓
3. Were the participants in the study representative of those who would be eligible for the test/service/intervention in the general or clinical population of interest?	✓	✓	✓	✓	✓	✓	✓
4. Were all eligible participants that met the prespecified entry criteria enrolled?	✓	✓	✓	✓	✓	✓	✓
5. Was the sample size sufficiently large to provide confidence in the findings?	Unknown	✓	✓	✓	✓	✓	Unknown
6. Was the test/service/intervention clearly described and delivered consistently across the study population?	✓	✓	✓	✓	✓	✓	✓
7. Were the outcome measures prespecified, clearly defined, valid, reliable, and assessed consistently across all study participants?	✓	✓	✓	✓	Partially	✓	✓
8. Were the people assessing the outcomes blinded to the participants' exposures/interventions?	✘	✘	✘	✘	✘	✘	✘
9. Was the loss to follow-up after baseline 20% or less? Were those lost to follow-up accounted for in the analysis?	✓	✓	✓	✓	✓	✓	✓
10. Did the statistical methods examine changes in outcome measures from before to after the intervention? Were statistical tests done that provided p values for the pre-to-post changes?	NA	NA	NA	NA	NA	NA	NA
11. Were outcome measures of interest taken multiple times before the intervention and multiple times after the intervention (i.e., did they use an interrupted time-series design)?	✘	✓	✓	✓	✓	✓	✘
12. If the intervention was conducted at a group level (e.g., a whole hospital, a community, etc.) did the statistical analysis take into account the use of individual-level data to determine effects at the group level?	Partially	Partially	Partially	Partially	Partially	Partially	Partially
**Overall rating**	**Fair** (main problem: the cohort sizes are absent)	**Good**	**Good**	**Good**	**Good**	**Good**	**Fair** (main problem: the cohort sizes are absent)
**Source**	Australian Government (2021)	Statistics Austria (2023)	Institut National de Criminalistique (2012)	Conselho Nacional De Justica Brazil (2020)	Stewart, Wilton, Baglole, & Miller (2019)	Tomášek and Rozum (2018)	Gendarmería de Chile (2016)


Criterion	Country					
	Denmark		Estonia	Finland	France	Ireland, Republic of
1. Was the study question or objective clearly stated?	✓	✓	✓	✓	✓	✓
2. Were eligibility/selection criteria for the study population prespecified and clearly described?	✓	✓	✓	✓	✓	✓
3. Were the participants in the study representative of those who would be eligible for the test/service/intervention in the general or clinical population of interest?	✓	✓	✓	✓	✓	✓
4. Were all eligible participants that met the prespecified entry criteria enrolled?	✓	✓	✓	✓	✓	✓
5. Was the sample size sufficiently large to provide confidence in the findings?	✓	✓	Unknown	✓	Unknown	✓
6. Was the test/service/intervention clearly described and delivered consistently across the study population?	✓	✓	✓	✓	✓	✓
7. Were the outcome measures prespecified, clearly defined, valid, reliable, and assessed consistently across all study participants?	✓	✓	✓	✓	✓	✓
8. Were the people assessing the outcomes blinded to the participants' exposures/interventions?	✘	✘	✘	✘	✘	✘
9. Was the loss to follow-up after baseline 20% or less? Were those lost to follow-up accounted for in the analysis?	✓	✓	✓	✓	✓	✓
10. Did the statistical methods examine changes in outcome measures from before to after the intervention? Were statistical tests done that provided *p* values for the pre-to-post changes?	NA	NA	NA	NA	NA	NA
11. Were outcome measures of interest taken multiple times before the intervention and multiple times after the intervention (i.e., did they use an interrupted time-series design)?	✘	✓	✘	✘	✓	✓
12. If the intervention was conducted at a group level (e.g., a whole hospital, a community, etc.) did the statistical analysis take into account the use of individual-level data to determine effects at the group level?	✘	Partially	✘	✘	✘	Partially
**Overall rating**	**Fair** (main problem: few details about methods and data source)	**Good**	**Fair** (main problems: no cohort sizes, no detailed breakdown of the cohort)	**Fair** (main problem: few details about methods and data source)	**Fair** (main problem: no cohort sizes)	**Good**
**Source**	Kristoffersen (2022)	Statistics Denmark (2023)	Ahven et al. (2019)	Kristoffersen (2022)	Ministère de la Justice (2022)	Central Statistics Office Ireland, 2022a, Central Statistics Office Ireland, 2022b


Criterion	Country					
	Iceland	Israel	Jamaica	Japan	Korea, Republic of	Malaysia
1. Was the study question or objective clearly stated?	✓	✓	✓	✘	✘	✘
2. Were eligibility/selection criteria for the study population prespecified and clearly described?	✓	✓	✓	✓	✓	✓
3. Were the participants in the study representative of those who would be eligible for the test/service/intervention in the general or clinical population of interest?	✓	✓	✓	✓	✓	✓
4. Were all eligible participants that met the prespecified entry criteria enrolled?	✓	✓	✓	Unknown	Unknown	Unknown
5. Was the sample size sufficiently large to provide confidence in the findings?	✓	✓	✓	✓	✓	✓
6. Was the test/service/intervention clearly described and delivered consistently across the study population?	✓	✓	✓	✓	✓	✓
7. Were the outcome measures prespecified, clearly defined, valid, reliable, and assessed consistently across all study participants?	✓	✓	✓	✓	✓	✓
8. Were the people assessing the outcomes blinded to the participants' exposures/interventions?	✘	✘	✘	✘	✘	✘
9. Was the loss to follow-up after baseline 20% or less? Were those lost to follow-up accounted for in the analysis?	✓	✓	✓	✓	✓	✓
10. Did the statistical methods examine changes in outcome measures from before to after the intervention? Were statistical tests done that provided *p* values for the pre-to-post changes?	NA	NA	NA	NA	NA	NA
11. Were outcome measures of interest taken multiple times before the intervention and multiple times after the intervention (i.e., did they use an interrupted time-series design)?	✘	✓	✘	✘	✓	✓
12. If the intervention was conducted at a group level (e.g., a whole hospital, a community, etc.) did the statistical analysis take into account the use of individual-level data to determine effects at the group level?	✘	Partially	✘	✘	✘	✘
**Overall rating**	**Fair** (main problem: few details about methods and data source)	**Good**	**Fair** (main problems: no subgroup analyses, lack of data)	**Poor** (excerpt from conference proceedings, few details)	**Fair** (main problems: no subgroup analyses, lack of data)	**Fair** (main problems: no subgroup analyses, lack of data)
**Source**	Kristoffersen (2022)	Vaknin & Ben-Zvi (2021)	Department of Correctional Services (2017)	Morgan and Morgan (2019)	Korean Government (2022)	Wahab (2019)


Criterion	Country				
	Netherlands	New Zealand	Norway	Poland	the Philippines
1. Was the study question or objective clearly stated?	✓	✓	✓	✓	✓
2. Were eligibility/selection criteria for the study population prespecified and clearly described?	✓	✓	✓	✓	✓
3. Were the participants in the study representative of those who would be eligible for the test/service/intervention in the general or clinical population of interest?	✓	✓	✓	✓	✓
4. Were all eligible participants that met the prespecified entry criteria enrolled?	✓	✓	✓	✓	✓
5. Was the sample size sufficiently large to provide confidence in the findings?	✓	Unknown	✓	✓	✓
6. Was the test/service/intervention clearly described and delivered consistently across the study population?	✓	✓	✓	✓	✓
7. Were the outcome measures prespecified, clearly defined, valid, reliable, and assessed consistently across all study participants?	✓	✓	✓	✓	✓
8. Were the people assessing the outcomes blinded to the participants' exposures/interventions?	✘	✘	✘	✘	✘
9. Was the loss to follow-up after baseline 20% or less? Were those lost to follow-up accounted for in the analysis?	✓	✓	✓	✓	✓
10. Did the statistical methods examine changes in outcome measures from before to after the intervention? Were statistical tests done that provided *p* values for the pre-to-post changes?	NA	NA	NA	NA	NA
11. Were outcome measures of interest taken multiple times before the intervention and multiple times after the intervention (i.e., did they use an interrupted time-series design)?	✓	✘	✘	✓	✘
12. If the intervention was conducted at a group level (e.g., a whole hospital, a community, etc.) did the statistical analysis take into account the use of individual-level data to determine effects at the group level?	Partially	Partially	✘	Partially	✘
**Overall rating**	**Good**	**Fair** (main problem: the cohort sizes are absent)	**Fair** (main problem: few details about methods and data source)	**Good**	**Fair** (main problems: lack of data)
**Source**	Ministry of Justice (2017)	Department of Corrections (2022)	Kristoffersen (2022)	Jaki (2018)	Not published. Obtained from Bureau of Criminal Justice and Penology of the Philippines


Criterion	Country					
	Sweden		Switzerland	Taiwan	Thailand	UK: England and Wales
1. Was the study question or objective clearly stated?	✓	✓	✓	✓	✘	✓
2. Were eligibility/selection criteria for the study population prespecified and clearly described?	✓	✓	✓	✓	✓	✓
3. Were the participants in the study representative of those who would be eligible for the test/service/intervention in the general or clinical population of interest?	✓	✓	✓	✓	✓	✓
4. Were all eligible participants that met the prespecified entry criteria enrolled?	✓	✓	✓	✓	Unknown	✓
5. Was the sample size sufficiently large to provide confidence in the findings?	✓	✓	✓	Unknown	Unknown	✓
6. Was the test/service/intervention clearly described and delivered consistently across the study population?	✓	✓	✓	✓	✓	✓
7. Were the outcome measures prespecified, clearly defined, valid, reliable, and assessed consistently across all study participants?	✓	✓	✓	✓	✓	✓
8. Were the people assessing the outcomes blinded to the participants' exposures/interventions?	✘	✘	✘	✘	✘	✘
9. Was the loss to follow-up after baseline 20% or less? Were those lost to follow-up accounted for in the analysis?	✓	✓	✓	✓	✓	✓
10. Did the statistical methods examine changes in outcome measures from before to after the intervention? Were statistical tests done that provided *p* values for the pre-to-post changes?	NA	NA	NA	NA	NA	NA
11. Were outcome measures of interest taken multiple times before the intervention and multiple times after the intervention (i.e., did they use an interrupted time-series design)?	✘	✓	✘	✘	✘	✘
12. If the intervention was conducted at a group level (e.g., a whole hospital, a community, etc.) did the statistical analysis take into account the use of individual-level data to determine effects at the group level?	✘	Partially	Partially	✘	✘	Partially
**Overall rating**	**Fair** (main problem: few details about methods, data source)	**Good**	**Good**	**Fair** (main problem: the cohort sizes are absent)	**Poor** (no supporting data provided)	**Good**
**Source**	Kristoffersen (2022)	National Council for Crime Prevention (2022)	Federal Statistical Office (2018)	Tsai and Wu (2022)	Thailand Institute of Justice (2021)	Ministry of Justice (2022)


Criterion	Country		
	UK: Northern Ireland	UK: Scotland	USA (34 states)
1. Was the study question or objective clearly stated?	✓	✓	✓
2. Were eligibility/selection criteria for the study population prespecified and clearly described?	✓	✓	✓
3. Were the participants in the study representative of those who would be eligible for the test/service/intervention in the general or clinical population of interest?	✓	✓	✓
4. Were all eligible participants that met the prespecified entry criteria enrolled?	✓	✓	✓
5. Was the sample size sufficiently large to provide confidence in the findings?	✓	✓	✓
6. Was the test/service/intervention clearly described and delivered consistently across the study population?	✓	✓	✓
7. Were the outcome measures prespecified, clearly defined, valid, reliable, and assessed consistently across all study participants?	✓	✓	✓
8. Were the people assessing the outcomes blinded to the participants' exposures/interventions?	✘	✘	✘
9. Was the loss to follow-up after baseline 20% or less? Were those lost to follow-up accounted for in the analysis?	✓	✓	✓
10. Did the statistical methods examine changes in outcome measures from before to after the intervention? Were statistical tests done that provided *p* values for the pre-to-post changes?	NA	NA	NA
11. Were outcome measures of interest taken multiple times before the intervention and multiple times after the intervention (i.e., did they use an interrupted time-series design)?	✘	✘	✓
12. If the intervention was conducted at a group level (e.g., a whole hospital, a community, etc.) did the statistical analysis take into account the use of individual-level data to determine effects at the group level?	Partially	Partially	Partially
**Overall rating**	**Good**	**Good**	**Good**
**Source**	Department of Justice (2021)	Scottish Government (2021)	Bureau of Justice Statistics (2021)

## Appendix A Terms and search conditions used for systematic search in publication databases

Search on SAGE, MEDLINE, PsychINFO, EMBASE, PsycARTICLES, and Web Science from January 1, 2019, to February 2, 2023, with no language restrictions: prisoners AND (prevalence OR rate*) AND (recidivis* OR reoffend* OR reconviction) AND (Algeria OR Argentina OR Australia OR Bangladesh OR Brazil OR Cambodia OR Canada OR Chile OR China OR Colombia OR Cuba OR Egypt OR “El Salvador” OR Ethiopia OR France OR Germany OR India OR Indonesia OR Iran OR Iraq OR Italy OR Japan OR Kazakhstan OR Kenya OR Malaysia OR Mexico OR Morocco OR (Myanmar OR Burma) OR Nigeria OR Pakistan OR Peru OR Philippines OR Poland OR “South Korea” OR Russia* OR Rwanda OR Saudi Arabia OR South Africa OR Spain OR Taiwan OR Tanzania OR Thailand OR Turkey OR Turkmenistan OR Uganda OR Ukraine OR (“United Kingdom” OR “UK” OR “England and Wales”) OR (“United States of America” OR “United States” OR USA) OR Venezuela OR Vietnam).

## Appendix C Identified studies and reports that did not satisfy the inclusion criteria

The studies used cross-sectional data to estimate recidivism in prisoners. The most common definition – the prevalence of individuals that were sentenced for the second or more time among all sentenced individuals in a given reporting period.

## Appendix G Supplementary data

Supplementary material 1 Appendices Supplementary material 1

Supplementary material 2 information vignettes for recidivism rates for individual countries Supplementary material 2

## References

[bb0155] Ahven A., Tamm K., Sööt M.-L. (2019). Kuritegevus Eestis 2019: Retsidiivsus. Ministry of Justice,  Estonia, Tallin. https://www.kriminaalpoliitika.ee/sites/krimipoliitika/files/elfinder/dokumendid/kuritegevus_9_kuud_2020.pdf.

[bb0005] Andersen S.N., Skardhamar T. (2017). Pick a number: Mapping recidivism measures and their consequences. Crime & Delinquency.

[bb0010] Anser M.K., Yousaf Z., Nassani A.A., Alotaibi S.M., Kabbani A., Zaman K. (2020). Dynamic linkages between poverty, inequality, crime, and social expenditures in a panel of 16 countries: Two-step GMM estimates. Journal of Economic Structures.

[bb0015] Antwi A. (2015). https://ugspace.ug.edu.gh/bitstream/handle/123456789/8364/Alex%20Antwi%20_%20Social%20Reintegration%20of%20Offenders%20and%20Recidivism%20in%20Ghana_2015.pdf.

[bb0020] Australian Government (2021). Report on Government Services 2021: C Justice.

[bb0255] Balduzzi S., Rücker G., Schwarzer G. (2019). How to perform a meta-analysis with R: a practical tutorial. Evidence-Based Mental Health.

[bb0025] BBC News (2019). https://www.bbc.co.uk/news/stories-48885846.

[bb0035] Browne R. (2020). *but they seem to work*. Yle.

[bb0335] Bureau of Justice Statistics (2021). https://bjs.ojp.gov/sites/g/files/xyckuh236/files/media/document/rpr34s125yfup1217.pdf.

[bb0030] Bergman M., Seepersad R., Safranoff A., Cafferata F. (2020). Center for Latin American Studies on Insecurity and Violence.

[bb0045] Camilleri M.R. (2016). https://www.um.edu.mt/library/oar/handle/123456789/19118.

[bb0050] Central Statistics Office Ireland (2022). Prison Re-offending Statistics 2019. https://www.cso.ie/en/releasesandpublications/ep/p-pros/prisonre-offendingstatistics2019/detailsof1-yearcustodialre-offending/.

[bb0055] Central Statistics Office Ireland (2022). Probation Re-Offending Statistics 2018. https://www.cso.ie/en/releasesandpublications/ep/p-prs/probationre-offendingstatistics2018/.

[bb0065] Conselho Nacional De Justica Brazil (2020). https://www.cnj.jus.br/wp-content/uploads/2020/03/Panorama-das-Reentradas-no-Sistema-Socioeducativo.pdf.

[bb0070] Corporación Excelencia en la Justicia (2023). Reincidencia carcelaria en Colombia..

[bb0080] Department of Correctional Services (2017). Improving prisons and reducing recidivism armed with data and information.

[bb0085] Department of Corrections (2022). https://www.corrections.govt.nz/__data/assets/pdf_file/0010/44398/Annual_Report_2020_2021_Final_Web.pdf.

[bb0090] Department of Justice (2021). Adult and youth reoffending in Northern Ireland 2018-19 cohort.

[bb0095] Fazel S., Wolf A. (2015). A systematic review of criminal recidivism rates worldwide: Current difficulties and recommendations for best practice. PLoS One.

[bb0100] Federal Statistical Office (2015). https://www.bfs.admin.ch/bfs/en/home/statistics/crime-criminal-justice/recidivism/analysis.assetdetail.350339.html.

[bb0310] Federal Statistical Office (2018). https://www.bfs.admin.ch/bfs/en/home/statistics/crime-criminal-justice/recidivism.html.

[bb0105] Fiji Corrections Service (2021). https://www.corrections.gov.fj/wp-content/uploads/2021/11/FCS-2018-2019-ANNUAL-REPORT.pdf.

[bb0110] Gendarmería de Chile (2016). Reincidencia delictual en egresados del sistema penitenciario chileno año 2011.

[bb0115] Government of Ontario (2021). Rates of Recidivism in Ontario.

[bb0120] Graunbøl H.M., Kielstrup B., Muiluvuori M.-L., Tyni S., Baldursson E.S., Gudmundsdottir H., Lindstén K. (2010). Retur: En nordisk undersøgelse af recidiv blant klienter i kriminalforsorgen.

[bb0125] Gruszczyńska B., Gruszczyński M. (2023). Crime and punishment—Crime rates and prison population in Europe. SSRN.

[bb0135] Instituto Nacional de Estadística y Geografía (2016). https://www.inegi.org.mx/programas/enpol/2016/.

[bb0140] Jehle J., Albrecht H., Jehle J. (2014). National reconviction statistics and studies in Europe.

[bb0145] Kipena K., Zavackis A., Nikisins J. (2012). https://wp.unil.ch/space/files/2012/07/Latvian_Probation_Recidivism_Study_Summary.pdf.

[bb0150] Korean Government (2022). https://www.index.go.kr/unify/idx-info.do?idxCd=4267.

[bb0160] Kristoffersen R. (2022). Correctional Statistics of Denmark, Finland, Iceland, Norway and Sweden 2016–2020.

[bb0165] Lehti, M., Kivivuori, J., Bergsdóttir, G.S., Engvold, H., Granath, S., Jónasson, J.O., … Suonpää, K. (2019). *Nordic homicide report: homicide in Denmark, Finland, Iceland, Norway and Sweden, 2007–2016 (Number: 37/2019).* University of Helsinki, Institute of Criminology and Legal Policy, Finland, Helsinki. http://hdl.handle.net/10138/306217.

[bb0170] Mecanismo Nacional de Prevención de la Tortura (2019). Adelanto de datos - Anuario Estadístico de Personas Privadas de Libertad 2019.

[bb0175] Ministère de la Justice (2013). Mesurer la récidive: Contribution à la conférence de consensus de prévention de la récidive.

[bb0180] Ministère de la Justice (2022). https://www.justice.gouv.fr/sites/default/files/migrations/portail/art_pix/Infos_rapides_justice_n1.pdf.

[bb0130] Institut National de Criminalistique et Criminologie (2012). Wederopsluiting na vrijlating uit de gevangenis. National Institute for Criminalistics and Criminology, Belgium, Brussels. https://nicc.fgov.be/upload/publicaties/rapport_27.pdf.

[bb0185] Ministerio de Justicia y Derechos Humanos (2020). https://www.argentina.gob.ar/sites/default/files/2019/08/informeanualsentenciascondenatorias2020.pdf.

[bb0190] Ministerul Justitiei (2021). https://anp.gov.ro/wp-content/uploads/2021/12/SITUAŢIA-LUNARĂ-noiembrie-2021-site.pdf.

[bb0230] Ministry of Home Affairs (2020). https://ncrb.gov.in/sites/default/files/PSI_2020_as_on_27-12-2021_0.pdf.

[bb0195] Ministry of Justice (2017). Repris.

[bb0200] Ministry of Justice (2022). https://www.gov.uk/government/statistics/proven-reoffending-statistics-october-to-december-2020/proven-reoffending-statistics-october-to-september-2020.

[bb0205] Ministry of Justice (2023). https://www.gov.uk/government/collections/proven-reoffending-statistics.

[bb0220] Morgan I., Morgan N. (2019). https://apcca.org/pdf-template/?pdf=https://apcca.org/wp-content/uploads/2021/11/APCCA_Report_2019_FinalUlaanbaatar_Mongolia.pdf.

[bb0225] Mundia L., Matzin R., Mahalle S., Hamid M., Osman R. (2016). Recidivism in Brunei inmates – Estimating the rates and predicting reoffending. International Journal of Emergency Mental Health and Human Resilience.

[bb0300] National Council for Crime Prevention (2017). Recidivists among all initial events by principal sanction 2005–2009.

[bb0305] National Council for Crime Prevention (2022). https://bra.se/download/18.146acb6517fd55784014b76/1653979808086/Statistikrapport_aterfall_prel_2019.pdf.

[bb0235] NIH (2021). Study Quality Assessment Tools.

[bb0245] Petersilia J. (2011). Beyond the prison bubble. The Wilson Quarterly (1976).

[bb0250] Robinson C., Sorbie A., Huber J., Teasdale J., Scott K., Purver M., Elliott I. (2021). https://assets.publishing.service.gov.uk/media/601980c8e90e07128d62cd64/RESOLVE_report.pdf.

[bb0210] Jaki P. (2018). https://orka2.sejm.gov.pl/INT8.nsf/klucz/ATTB68JU5/%24FILE/i26703-o1.pdf.

[bb0260] Scottish Government. (2021). Reconviction rates in Scotland: 2018-19 offender cohort. https://www.gov.scot/publications/reconviction-rates-scotland-2018-19-offender-cohort/#:∼:text=The%20reconviction%20rate%2C%20which%20is,26.4%25%20in%202017%2D18.

[bb0265] Seabold S., Perktold J. (2010). In proceedings of the 9th Python in Science Conference.

[bb0240] SPAC (2018). https://www.nccourts.gov/assets/documents/publications/recidivism_2018.pdf.

[bb0270] SPS (2021). SPS Annual Statistics Release for 2021. Singapore Prison Service.

[bb0275] Statistics Austria (2023). Statcube. https://www.statistik.at/en/databases/statcube-statistical-database.

[bb0285] Statistics Bureau of Japan (2017). https://www.stat.go.jp/english/data/jinsui/2017np/index.html.

[bb0290] Statistics Denmark (2023). https://www.statbank.dk/.

[bb0295] Statistics Division Prison Headquarters (2021). http://prisons.gov.lk/web/wp-content/uploads/2021/05/prison-statistics-2021.pdf.

[bb0075] Stewart L.A., Wilton G., Baglole S., Miller R. (2019). https://www.csc-scc.gc.ca/005/008/092/005008-r426-en.pdf.

[bb0315] Thailand Institute of Justice (2016). A comparative study of treatment of prisoners and non-custodial measure in ACEAN.

[bb0320] Thailand Institute of Justice (2021). Research on the Causes of Recidivism in Thailand.

[bb0325] Tomášek J., Rozum J. (2018). Recidivism as a measure of the effectiveness of sanctions: Experience from the Czech Republic. AUC Philosophica et Historica.

[bb0330] Tsai I.-C., Wu Y.-T. (2022). Recent analysis on crime trends and observations on policy development in Taiwan. https://www.tpi.moj.gov.tw/media/208454/1090203-recent-analysis-on-crime-trends-and-observations-on-policy-development-in-taiwan.pdf?mediaDL=true.

[bb0340] United Nations Office on Drugs and Crime (2022). https://www.unodc.org/documents/data-and-analysis/prison/Pilot_prison_research_brief_2022.pdf.

[bb0345] Vaknin Y., Ben-Zvi K. (2021). https://www.gov.il/BlobFolder/dynamiccollectorresultitem/retsidivisem_2014-2019/he/דוח%20רצידיביזם%20של%20אסירים%20פליליים%202019-2014%20-%2008062021%20(1).pdf.

[bb0350] Wahab S.A. (2019). 170th International Training Course of UNAFEI.

[bb0355] Wolf A., Gray R., Fazel S. (2014). Violence as a public health problem: An ecological study of 169 countries. Social Science & Medicine.

[bb0360] World Prison Brief (2022). https://www.prisonstudies.org/highest-to-lowest/prison-population-total.

[bb0365] Yukhnenko D., Sridhar S., Fazel S. (2019). A systematic review of criminal recidivism rates worldwide: 3-year update. Wellcome Open Research.

[bb0370] Yukhnenko D., Wolf A., Blackwood N., Fazel S. (2019). Recidivism rates in individuals receiving community sentences: A systematic review. PLoS One.

