# Supplementary material for: Criminal recidivism rates globally: A 6-year systematic review update
Source: J Crim Justice. 2023 Sep-Oct;88:102115. doi: 10.1016/j.jcrimjus.2023.102115 (PMC10933794; doi:10.1016/j.jcrimjus.2023.102115)
Supplement: Supplementary material 2 — information vignettes for recidivism rates for individual countries [file mmc2.pdf]

Australia

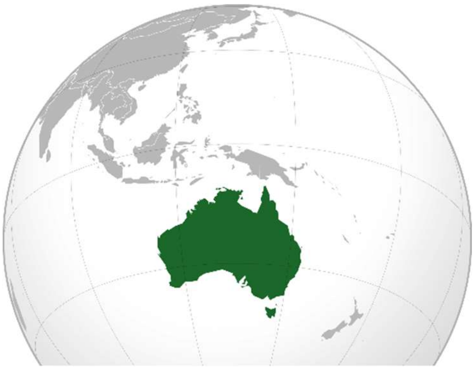

Reporting period
2019-2020

Relevant statistics for the country (in 2021)
Population: 25,766,605 (Australian Bureau of Statistics, 2022)
Average prison population: 42,203 (World Prison Brief, 2022)
Imprisonment rate: 165 per 100,000 (World Prison Brief, 2022)

Sources for criminal recidivism data
Australian Government Productivity Commission (2021). Report on Government Services 2021. URL

Reporting agencies
Australian Government Productivity Commission URL

Population
☑ Released prisoners
☑ Community-sentenced individuals

Reported outcomes
☑ Reconviction
☐ Re-arrest
☑ Reimprisonment
☐ Other (…)

Follow-up periods
☐ 1 year
☑ 2 years
☐ 3 years
☐ Other (…)

Cohort sizes
Released prisoners: NA
Community-sentenced individuals*: NA
**Individuals starting community order or suspended sentence order*

Recidivism rates
2-year reconviction in released prisoners: 54.9%
2-year re-imprisonment in released prisoners: 46.0%
2-year reconviction in community-sentenced individuals: 16.1%

Outcome definition
- Reconviction: return to corrective services (prison or community corrections orders) for those who were released from prison. For those released from community-sentences, reconviction is receiving a new community order.
- Re-imprisonment: return to prison for those who were released from prison or from community corrections orders.

Factors contributing to the recidivism rate estimates
- COVID-19 may have impacted service delivery by correctional services as well as data collection and reporting. The number of people entering and exiting prisons in the last 4 months of the reporting period was also reduced due to the pandemic (Australian Government Productivity Commission, 2021).

Notes on recidivism rates trends
- Recidivism rates are slightly higher than the previous 2-year recidivism report (2-year reconviction in released prisoners: 51.3%, 2-year re-imprisonment in released prisoners: 44.5%, 2-year reconviction in community-sentenced individuals: 15%) (Australian Government Productivity Commission, 2021).
- The number of drug arrests went up form 84,384 in 2010-2011 to 166,321 in 2019-2020. Most arrests were associated drug consumption, not distribution. The proportion of amphetamine related arrests also increased from 2010 to 2020. Most of the arrests were associated with cannabis (Australian Institute of Health and Welfare, 2023)
- ‘Illicit drug offences remained steady from 2010–11 to 2018–19 before dropping in 2019–20 (45,252) and rising again in 2020–21 (50,958)’ (Australian Institute of Health and Welfare, 2023).

References
Australian Bureau of Statistics. (2022). National, state and territory population.

https://www.abs.gov.au/statistics/people/population/national-state-and-territory-population/dec-2021
Australian Government Productivity Commission. (2021). *Report on Government Services 2021*. https://www.pc.gov.au/ongoing/report-on-government-services/2021/justice
Australian Institute of Health and Welfare. (2023). *Alcohol, tobacco & other drugs in Australia*. Retrieved 01/07/2023 from https://www.aihw.gov.au/reports/alcohol/alcohol-tobacco-other-drugs-australia/contents/priority-populations/people-in-contact-with-the-criminal-justice-system
World Prison Brief. (2022). *Australia*. Retrieved 04/05/2023 from https://www.prisonstudies.org/country/australia

Austria

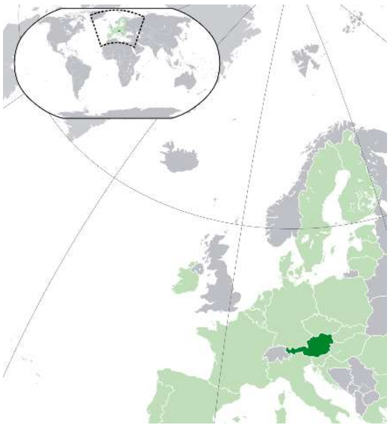

Reporting period
2017

Relevant statistics for the country (in 2017-2018)
Population: 8,819,901 (Wordometer, 2023)
Average prison population: 8,692 (World Prison Brief, 2023)
Imprisonment rate: 98 per 100,000 (World Prison Brief, 2023)
Homicide rate: 0.7 per 100,000 (Bundeskriminalamt, 2018)
Robbery rate: 24.1 per 100,000 (Federal Ministry Republic of Austria, 2019)
GDP per capita: 47,429 (World Bank, 2023)

Sources for criminal recidivism data
Statistics Austria (2022). Statistics on convictions and reconvictions. URL
Statistics Austria (2022). STATcube. URL

Reporting agencies
Statistics Austria URL

Population
☑ Released prisoners
☑ Community-sentenced individuals

Reported outcomes
☑ Reconviction
☐ Re-arrest
☐ Reimprisonment
☑ Other (…)

Follow-up periods
☑ 1 year
☑ 2 years
☑ 3 years
☑ Other (4 years, 4-5 years)

Cohort sizes
Released prisoners: 6,607
Released prisoners (STATcube): 3,819
Community-sentenced individuals*: 10,636
Community-sentenced individuals* (STATcube): 6,652

**Individuals starting a new sentence. Includes conditional imprisonment*

Recidivism rates
1-year reconviction in released prisoners: 13.5%
2-year reconviction in released prisoners: 24.3%
3-year reconviction in released prisoners: 30.3%
4-year reconviction in released prisoners: 34.7%
1-year reconviction in non-custodial sentences (excluding fines): 11.2%
2-year reconviction in non-custodial sentences (excluding fines): 21.3%
3-year reconviction in non-custodial sentences (excluding fines): 27.3%
4-year reconviction in non-custodial sentences (excluding fines): 31.6%
STATCube data:
4-5 year re-imprisonment in released prisoners: 46.6%
4-5 year reconviction in non-custodial sentences (excluding fines): 38.0%

Outcome definition

- Reconviction: 'Legally effective reconvictions within four years after the base event. The base event is either the release from prison or institutional confinement, or the legally effective conviction to something other than unconditional prison or institutional confinement' (Statistics Austria, 2023b).

Factors contributing to the recidivism rate estimates

- The cohort sizes and rates vary depending on the source of data 'Statistics on convictions and reconvictions' (Statistics Austria, 2023b) vs. STATCube (Statistics Austria, 2023a). This might be due to different data linkage, index sentence operationalisation, and the specific way of data extracted by the end user.

Notes on recidivism rates trends

- The number of reported drug crimes increased from 26,892 in 2011 to 42,610 in 2017. Then remained relatively stable from 2017 to 2020 (Federal Ministry Republic of Austria, 2021).

- COVID-19 led to ‘somewhat fewer’ reported drug crimes in 2020. After the restrictions were lifted, the trend reversed back (Federal Ministry Republic of Austria, 2021).

References

Bundeskriminalamt. (2018). *Polizeiliche Kriminalstatistik 2017*. https://bundeskriminalamt.at/501/files/PKS_17_Broschuere_Web.pdf

Federal Ministry Republic of Austria. (2019). *Crime Statistics 2018 - Conclusions*. https://bundeskriminalamt.at/501/files/KrimStat_Fazit_ENGLISCH_V20190514.pdf

Federal Ministry Republic of Austria. (2021). *Drug-Related Crime Annual Report 2020*. https://bundeskriminalamt.at/302/files/Suchtmittel_2020_engl_web_20210721.pdf

Statistics Austria. (2023a). *Statcube*. https://www.statistik.at/en/databases/statcube-statistical-database

Statistics Austria. (2023b). *Statistics on convictions and reconvictions*. Retrieved 01/07/2023 from https://www.statistik.at/en/statistics/population-and-society/criminality-and-security/statistics-on-convictions-and-reconvictions

Wordometer. (2023). *Austria Population*. Retrieved 01/07/2023 from https://www.worldometers.info/world-population/austria-population/

World Bank. (2023). *GDP per capita - Austria*. Retrieved 05/06/2023 from https://data.worldbank.org/indicator/NY.GDP.PCAP.CD?locations=AT

World Prison Brief. (2023). *Austria*. Retrieved 04/05/2023 from https://www.prisonstudies.org/country/austria

Belgium

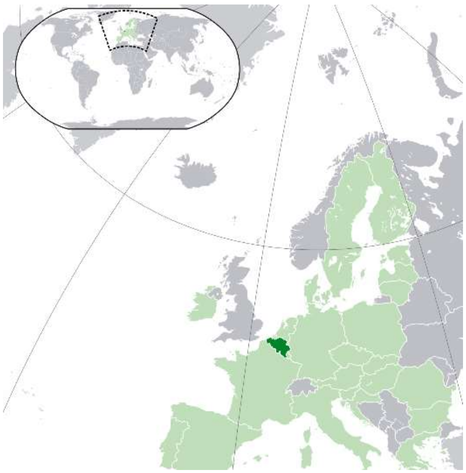

Reporting period
2003-2005

Relevant statistics for the country (in 2004)
Population: 10,445,852 (Statbel, 2022)
Average prison population: 9,245 (World Prison Brief, 2021)
Imprisonment rate: 89 per 100,000 (World Prison Brief, 2021)

Sources for criminal recidivism data
Institut National de Criminalistique et Criminologie (2012). Wederopsluiting na vrijlating uit de gevangenis. URL

Reporting agencies
Institut National de Criminalistique et Criminologie URL

Population
☑ Released prisoners
☐ Community-sentenced individuals

Reported outcomes
☐ Reconviction
☐ Re-arrest
☑ Reimprisonment
☐ Other (…)

Follow-up periods
☐ 1 year
☐ 2 years
☐ 3 years
☑ 5 years

Cohort sizes
Released prisoners*: 1,175
**Individuals starting a new sentence*

Recidivism rates
5-year re-imprisonment in released prisoners: 62.3%

Outcome definition

- Re-imprisonment: those who were released from prison who returned to prison within 5-years (Institut National de Criminalistique et Criminologie, 2012)

References
Institut National de Criminalistique et Criminologie. (2012). Wederopsluiting na vrijlating uit de gevangenis. https://nicc.fgov.be/upload/publicaties/rapport_27.pdf
Statbel. (2022). *Structure of the Population*. https://statbel.fgov.be/en/themes/population/structure-population#panel-13
World Prison Brief. (2021). *Belgium*. Retrieved 04/05/2023 from https://www.prisonstudies.org/country/belgium

Brazil

Reporting period
2015

Relevant statistics for the country (in 2016)
Population: 205,157,000 (Country Economy, 2016)
Average prison population: 722,120 (World Prison Brief, 2021)
Imprisonment rate: 349 per 100,000 (World Prison Brief, 2021)

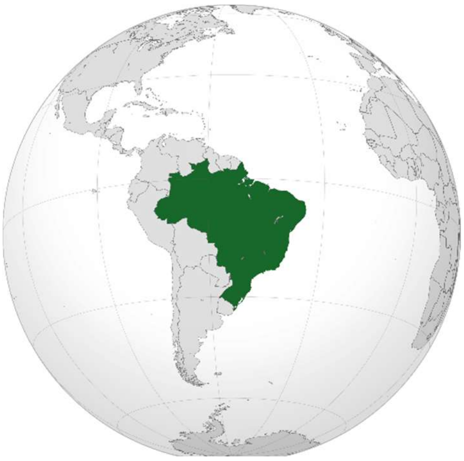

Sources for criminal recidivism data
Conselho Nacional De Justica (2020). Reentradas e reiterações infracionais um olhar sobre os sistemas socioeducativo e prisional brasileiros. URL

Reporting agencies
Conselho Nacional De Justica URL

Population
☑ Released prisoners
☐ Community-sentenced individuals

Reported outcomes
☐ Reconviction
☐ Re-arrest
☑ Reimprisonment
☐ Other (…)

Follow-up periods
☐ 1 year
☐ 2 years
☐ 3 years
☑ Other (4 years)

Cohort sizes
Released prisoners: NA

Recidivism rates
4-year re-imprisonment in released prisoners: 42.5%
4-year reconviction to the socio-educational system: 23.9%

Outcome definition
- Recidivism is defined as reimprisonment: re-entering either the prison system or the socio-educational system within 4 years of being released from either (Conselho Nacional De Justica Brazil, 2020).

References
Conselho Nacional De Justica Brazil. (2020). *Reentradas e reiterações infracionais um olhar sobre os sistemas socioeducativo e prisional brasileiros*. https://www.cnj.jus.br/wp-content/uploads/2020/03/Panorama-das-Reentradas-no-Sistema-Socioeducativo.pdf
Country Economy. (2016). *Brazil - Population*. Retrieved 04/06/2021 from https://countryeconomy.com/demography/population/brazil
World Prison Brief. (2021). *Brazil*. Retrieved 04/05/2023 from https://www.prisonstudies.org/country/Brazil

Canada (federal)

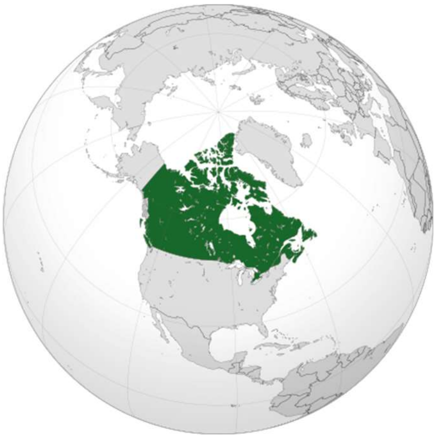

Reporting period
2011-2012

Relevant statistics for the country
Population:
Average prison population: 40,663 (World Prison Brief, 2020)
Imprisonment rate: 117 per 100,000 (World Prison Brief, 2020)
Gini index: 1.7 (World Bank, 2020)
Homicide rate: 1.7 per 100,000 (The Global Economy, 2023a)
Robbery rate: 86 per 100,000 (The Global Economy, 2023b)
GDP per capita: 52,224 (World Bank, 2023)

Sources for criminal recidivism data
Correctional Service Canada (2019). A Comprehensive Study of Recidivism Rates among Canadian Federal Offenders. URL

Reporting agencies
Correctional Service Canada URL

Population
☑ Released prisoners
☐ Community-sentenced individuals

Reported outcomes
☐ Reconviction
☐ Re-arrest
☐ Reimprisonment
☑ Reoffending

Follow-up periods
☑ 1 year
☑ 2 years
☑ 3 years
☑ 5 years

Population
☑ Released prisoners
☐ Community-sentenced individuals

Reported outcomes
☑ Reconviction
☐ Re-arrest
☐ Reimprisonment
☐ Other (…)

Follow-up periods
☑ 1 year
☑ 2 years
☑ 3 years
☑ Other (5 years)

Cohort sizes
Released federal prisoners: 8,893

Recidivism rates
1-year re-offending rate for federal prisoners: 17.1%
2-year re-offending rate for federal prisoners: 27.9%
3-year re-offending rate for federal prisoners: 35.4%
5-year re-offending rate for federal prisoners: 43.2%

Outcome definition

- Reoffending: committing any new offence during the follow-up according to Canadian Police Information Centre (Correctional Service Canada, 2019).

References

Correctional Service Canada. (2019). *A Comprehensive Study of Recidivism Rates among Canadian Federal Offenders*. https://www.csc-scc.gc.ca/005/008/092/005008-r426-en.pdf

The Global Economy. (2023a). *Canada: Homicide rate*. https://www.theglobaleconomy.com/Canada/homicide_rate/

The Global Economy. (2023b). *Canada: Robbery rate*. https://www.theglobaleconomy.com/Canada/robery/

World Bank. (2020). *Gini index - Canada*. https://data.worldbank.org/indicator/SI.POV.GINI?locations=CA

World Bank. (2023). *GDP per capita (current US$) - Canada*. https://data.worldbank.org/indicator/NY.GDP.PCAP.CD?locations=CA

World Prison Brief. (2020). *Canada*. Retrieved 04/05/2023 from https://www.prisonstudies.org/country/canada

Chile

Reporting period
2011

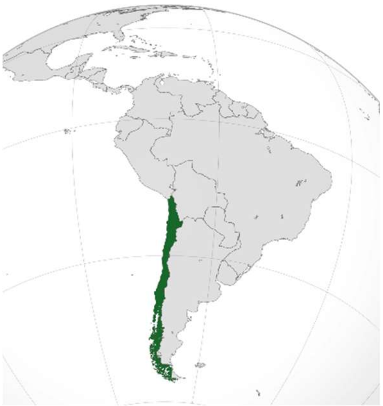

Relevant statistics for the country (in 2010)
Population: 17,004,162 (The World Bank, 2021)
Average prison population: 54,628 (World Prison Brief, 2023)
Imprisonment rate: 320 per 100,000 (World Prison Brief, 2023)
Gini index: 46.0 (World Bank, 2020)
Homicide rate: 3.7 per 100,000 (The Global Economy, 2018a)
Robbery rate: 535 per 100,000 (The Global Economy, 2018b)
GDP per capita: 14,629 (World Bank, 2023)

Sources for criminal recidivism data
Gendarmería de chile (2016). Unidad de estudios en criminología e innovación penitenciaria URL

Reporting agencies
Gendarmería de chile. URL

Population
☑ Released prisoners
☑ Community-sentenced individuals

Reported outcomes
☑ Reconviction
☐ Re-arrest
☐ Reimprisonment
☐ Other (…)

Follow-up periods
☐ 1 year
☑ 2 years
☐ 3 years
☐ 5 years

Cohort sizes
Released prisoners: NA
Community-sentenced individuals*: NA
**Individuals starting community order or suspended sentence order*

Recidivism rates
2-year recidivism rate in released prisoners: 39.08%
2-year recidivism rate among those with conditional liberty: 13.95%
2-year recidivism rate among those in the open subsystem (serving their sentence while free): 9.61%
2-year recidivism rate among those in the semi-open subsystem (serving sentences with leave from prison to work/receive training): 16.46%

Outcome definition

- Committing a new offence during the follow up (Gendarmería de Chile, 2016).

References

Gendarmería de Chile. (2016). *Unidad de estudios en criminología e innovación penitenciaria*. https://html.gendarmeria.gob.cl/doc/estadisticas/REINCIDENCIA_2011.pdf
The Global Economy. (2018a). *Chile: Homicide rate*. https://www.theglobaleconomy.com/Chile/homicide_rate/
The Global Economy. (2018b). *Chile: Robbery rate*. https://www.theglobaleconomy.com/Chile/robery/
The World Bank. (2021). *Population, total - Chile*. https://data.worldbank.org/indicator/SP.POP.TOTL?locations=CL
World Bank. (2020). *Gini index - Chile*. https://data.worldbank.org/indicator/SI.POV.GINI?locations=CL
World Bank. (2023). *GDP per capita (current US$) - Chile*. https://data.worldbank.org/indicator/NY.GDP.PCAP.CD?locations=CL
World Prison Brief. (2023). *Chile*. Retrieved 04/05/2023 from https://www.prisonstudies.org/country/Chile

Czech Republic

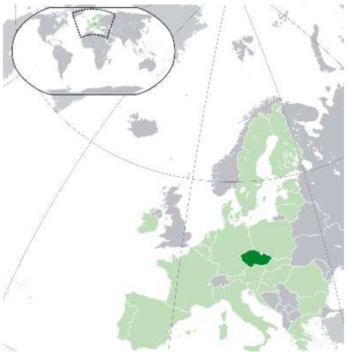

Reporting period
2012

Relevant statistics for the country (in 2012)
Population: 10,510,785 (The World Bank, 2021)
Average prison population: 22,641 (World Prison Brief, 2023)
Imprisonment rate: 215 per 100,000 (World Prison Brief, 2023)

Sources for criminal recidivism data

Tomášek, J. & Rozum, J. (2018). Recidivism as a Measure of the Effectiveness of Sanctions: Experience from the Czech Republic. URL

Reporting agencies
Institute of Criminology and Social Prevention URL

Population
☐ Released prisoners
☑ Community-sentenced individuals

Reported outcomes
☑ Reconviction
☐ Re-arrest
☐ Reimprisonment
☐ Other (…)

Follow-up periods
☐ 1 year
☑ 2 years
☐ 3 years
☐ Other (…)

Cohort sizes
Community-sentenced individuals*: 4,233
**Includes house arrest, community services, and a suspended sentence.*

Recidivism rates
2-year reconviction in community-sentenced individuals: 48.1 *
** Individuals starting a community sentence*

Outcome definition

- Being convicted of a new crime within 2 years of starting a community sentence.

References

The World Bank. (2021). *Population, total - Czechia*. https://data.worldbank.org/indicator/SP.POP.TOTL?locations=CZ
World Prison Brief. (2023). *Czech Republic*. Retrieved 04/05/2023 from https://www.prisonstudies.org/country/czech-republic

Denmark

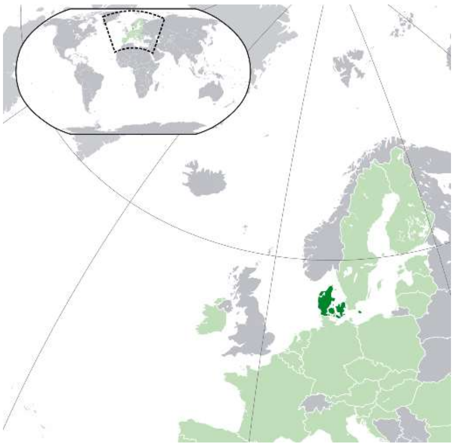

Reporting period
2018

Relevant statistics for the country (in 2018)
Population: 5,707,251 (Statistics Denmark, 2023a)
Average prison population: 3,421 (World Prison Brief, 2022)
Imprisonment rate: 60 per 100,000 (World Prison Brief, 2022)
Gini index: 28.2 (World Bank, 2020)
Homicide rate: 0.8 per 100,000 (Statistics Denmark, 2023b)
Robbery rate: 31.6 per 100,000 (Statistics Denmark, 2023b)
GDP per capita: 61,592 (World Bank, 2023)

Sources for criminal recidivism data
Kristoffersen, R. (2022). Correctional Statistics of Denmark, Finland, Iceland, Norway and Sweden 2016 – 2020. URL
Statistics Denmark. (2023b). Statbank Denmark Social Conditions. URL

Reporting agencies
University College of Norwegian Correctional Service URL
Statistics Denmark URL

Population
☑ Released prisoners
☐ Community-sentenced individuals

Reported outcomes
☑ Reconviction
☐ Re-arrest
☑ Reimprisonment
☐ Other (…)

Follow-up periods
☐ 1 year
☑ 2 years
☐ 3 years
☐ 5 years

Cohort sizes
Statistics Denmark:
Released prisoners: 2,710
Individuals given community sentences: 7,387
University College of Norwegian Correctional Service:
Released prisoners: 4,909

Recidivism rates
Statistics Denmark:
2-year reconviction rate in released prisoners: 66.0%
2-year reconviction rate in individuals given community sentences: 45.6%
University College of Norwegian Correctional Service:
2-year re-conviction rate in released prisoners: 32.0%

Outcome definition

- Re-conviction: new unconditional sentence within the follow-up period (Kristoffersen, 2022; Statistics Denmark, 2023c).

Factors contributing to the recidivism rate estimates

- The cohort sizes and rates vary depending on the source of data, University College of Norwegian Correctional Service (Kristoffersen, 2022) vs. Statistics Denmark (Statistics Denmark, 2023c). This might be due to different data linkage, index sentence operationalisation, and the specific way of data extracted by the end user.

References

Kristoffersen, R. (2022). *Correctional Statistics of Denmark, Finland, Iceland, Norway and Sweden 2016 – 2020.* https://www.fangelsi.is/media/almennt/Nordic-Statistics-2016_2020_final.pdf

Statistics Denmark. (2023a). *Population Figures*. https://www.dst.dk/en/Statistik/emner/borgere/befolkning/befolkningstal

Statistics Denmark. (2023b). *Reported criminal offences*. Retrieved 06/06/2023 from https://www.dst.dk/en/Statistik/emner/sociale-forhold/kriminalitet/anmeldte-forbrydelser

Statistics Denmark. (2023c). *Statbank Denmark Social Conditions*. https://www.statbank.dk/
World Bank. (2020). *Gini index - Denmark*. https://data.worldbank.org/indicator/SI.POV.GINI?locations=DK
World Bank. (2023). *GDP per capita (current US$) - Denmark*. https://data.worldbank.org/indicator/NY.GDP.PCAP.CD?locations=DK
World Prison Brief. (2022). *Denmark*. Retrieved 04/05/2023 from https://www.prisonstudies.org/country/denmark

Estonia

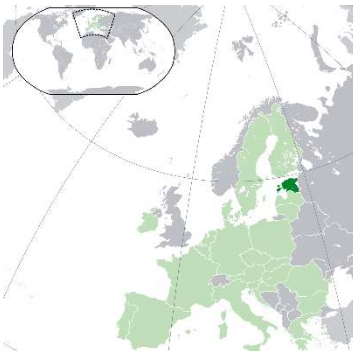

Reporting period
2015-2017

Relevant statistics for the country (in 2016)
Population: 1,315,944 (Statistics Estonia, 2023)
Average prison population: 2,681 (World Prison Brief, 2023)
Imprisonment rate: 204 per 100,000 (World Prison Brief, 2023)

Sources for criminal recidivism data
Kriminal Politikia (2019). Kuritegevus Eestis 2019, Retsidiivsus.
URL

Reporting agencies
Criminal Policy Department of the Ministry of Justice. URL

Population
☑ Released prisoners
☐ Community-sentenced individuals

Reported outcomes
☑ Reconviction
☐ Re-arrest
☐ Reimprisonment
☐ Other (…)

Follow-up periods
☐ 1 year
☑ 2 years
☐ 3 years
☐ Other (…)

Cohort sizes
Released prisoners: NA

Recidivism rates
2-year reconviction in released prisoners: 32%

Outcome definition
- Those who were reconvicted within 2 years of being released from prison or from starting a community sentence

References

Statistics Estonia. (2023). *Population*. Retrieved 06/06/2023 from https://www.stat.ee/en/find-statistics/statistics-theme/population
World Prison Brief. (2023). *Estonia*. Retrieved 04/05/2023 from https://www.prisonstudies.org/country/estonia

Finland

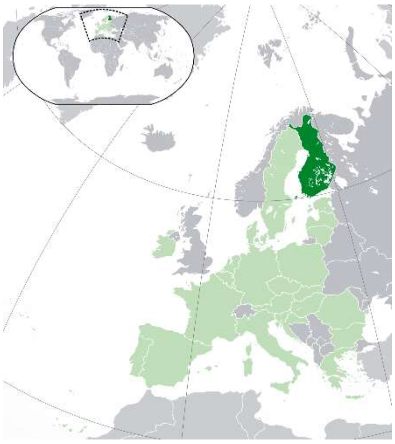

Reporting period
2018

Relevant statistics for the country (in 2018)
Population: 5,517,919 (Statistics Finland, 2019)
Average prison population: 2,910 (World Prison Brief, 2022)
Imprisonment rate: 53 per 100,000 (World Prison Brief, 2022)
Gini index: 27.3 (World Bank, 2020)
Homicide rate (in 2017): 1.2 per 100,000 (The Global Economy, 2023)
Robbery rate: 25.3 per 100,000 (Tilastokeskus, 2023)
GDP per capita: 49,988 (World Bank, 2023)

Sources for criminal recidivism data
Kristoffersen, R. (2022). Correctional Statistics of Denmark, Finland, Iceland, Norway and Sweden 2016 – 2020. URL

Reporting agencies
University College of Norwegian Correctional Service URL

Population	Reported outcomes	Follow-up periods
☑ Released prisoners	☑ Reconviction	☐ 1 year
☐ Community-sentenced individuals	☐ Re-arrest	☑ 2 years
	☐ Reimprisonment	☐ 3 years
	☐ Other (…)	☐ Other (…)

Cohort sizes
Released prisoners: 2,766

Recidivism rates
2-year reconviction in released prisoners: 33.0%

Outcome definition
- Reconviction: those released from prison who received a new conviction during the follow-up (Kristoffersen, 2022).

References

Kristoffersen, R. (2022). *Correctional Statistics of Denmark, Finland, Iceland, Norway and Sweden 2016 – 2020.* https://www.fangelsi.is/media/almennt/Nordic-Statistics-2016_2020_final.pdf
Statistics Finland. (2019). *Population growth smallest since 1970.* https://www.stat.fi/til/vaerak/2018/vaerak_2018_2019-03-29_tie_001_en.html
The Global Economy. (2023). *Finland: Homicide rate*. https://www.theglobaleconomy.com/Finland/homicide_rate/
Tilastokeskus. (2023). *Statistics on offences and coercive measures*. Retrieved 06/06/2023 from https://pxdata.stat.fi/PXWeb/pxweb/en/StatFin/StatFin__rpk/?tablelist=true
World Bank. (2020). *Gini index - Finland*. https://data.worldbank.org/indicator/SI.POV.GINI?locations=FI
World Bank. (2023). *GDP per capita (current US$) - Finland.* https://data.worldbank.org/indicator/NY.GDP.PCAP.CD?locations=FI
World Prison Brief. (2022). *Finland*. Retrieved 04/05/2023 from https://www.prisonstudies.org/country/finland

France

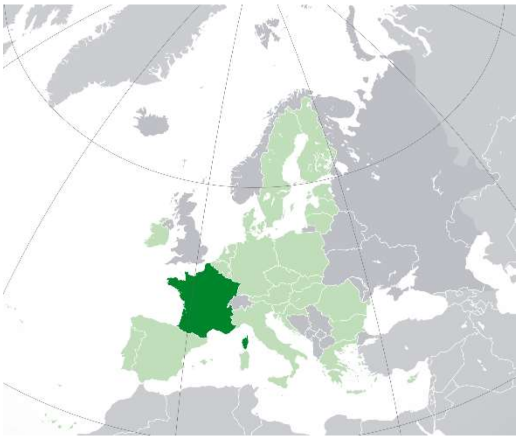

Reporting period
2016-2018

Relevant statistics for the country (in 2016)
Population: 66,991,000 (Institute National de La statistique et des etudes economiques, 2016)
Average prison population: 71, 190 (World Prison Brief, 2023)
Imprisonment rate: 110 per 100,000 (World Prison Brief, 2023)

Sources for criminal recidivism data
Ministere de la Justice (2022). Infos rapides justice. URL

Reporting agencies
Ministere de la Justice URL

Population
☑ Released prisoners
☐ Community-sentenced individuals

Reported outcomes
☐ Reconviction
☐ Re-arrest
☐ Reimprisonment
☑ Reoffending

Follow-up periods
☑ 1 year
☑ 2 years
☐ 3 years
☐ 5 years

Cohort sizes
Released prisoners: NA

Recidivism rates
1-year reoffending rate: 32.9%
2-year reoffending rate: 45.4%

Outcome definition
- Reconviction: a new offense often committed following release from prison (Ministère de la Justice, 2022).

References

Institute National de La statistique et des etudes economiques. (2016). *Bilan démographique 2016*. https://www.insee.fr/fr/statistiques/2554860
Ministère de la Justice. (2022). *Infos rapides justice*. https://www.justice.gouv.fr/sites/default/files/migrations/portail/art_pix/Infos_rapides_justice_n1.pdf
World Prison Brief. (2023). *France*. Retrieved 04/05/2023 from https://www.prisonstudies.org/country/france

Iceland

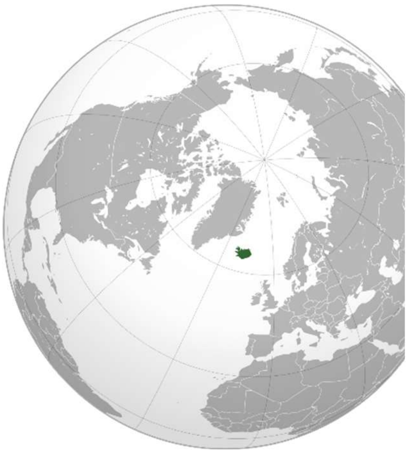

Reporting period
2018

Relevant statistics for the country (in 2018)
Population: 348,450 (Statistics Iceland, 2018)
Average prison population: 131 (World Prison Brief, 2022)
Imprisonment rate: 37 per 100,000 (World Prison Brief, 2022)
Gini index (in 2017): 26.1 (World Bank, 2018)
Homicide rate: 0.9 per 100,000 (Mactrotrends, 2021)
Robbery rate (in 2017): 14 per 100,000 (Mactrotrends, 2021)
GDP per capita: 74,461 (World Bank, 2023)

Sources for criminal recidivism data
Kristoffersen, R. (2022). Correctional Statistics of Denmark, Finland, Iceland, Norway and Sweden 2016 – 2020. URL

Reporting agencies
University College of Norwegian Correctional Service URL

Population
☑ Released prisoners
☐ Community-sentenced individuals

Reported outcomes
☑ Reconviction
☐ Re-arrest
☐ Reimprisonment
☐ Other (…)

Follow-up periods
☐ 1 year
☑ 2 years
☐ 3 years
☐ Other (…)

Cohort sizes
Released prisoners: 2,766

Recidivism rates
2-year reconviction in released prisoners: 33.0%

Outcome definition

- Reconviction: those released from prison who received a new conviction during the follow-up (Kristoffersen, 2022).

References

Kristoffersen, R. (2022). *Correctional Statistics of Denmark, Finland, Iceland, Norway and Sweden 2016 – 2020.* https://www.fangelsi.is/media/almennt/Nordic-Statistics-2016_2020_final.pdf
Mactrotrends. (2021). *Iceland Murder/Homicide Rate 1994-2023.* https://www.macrotrends.net/countries/ISL/iceland/murder-homicide-rate
Statistics Iceland. (2018). *Icelandic population grew by 3% last year*. https://www.statice.is/publications/news-archive/inhabitants/population-1st-of-january-2018/
World Bank. (2018). *Gini index - Iceland*. https://data.worldbank.org/indicator/SI.POV.GINI?locations=IS
World Bank. (2023). *GDP per capita (current US$) - Iceland.* https://data.worldbank.org/indicator/NY.GDP.PCAP.CD?locations=IS
World Prison Brief. (2022). *Iceland.* https://www.prisonstudies.org/country/iceland

Ireland

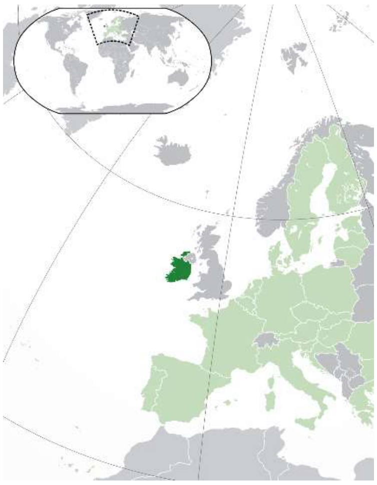

Reporting period
2018 - 2019

Relevant statistics for the country (in 2018)
Population: 4,857,000 (Central Statistics Office Ireland, 2018)
Average prison population: 3,893 (World Prison Brief, 2023)
Imprisonment rate: 80 per 100,000 (World Prison Brief, 2023)

Sources for criminal recidivism data

Central Statistics Office. (2022a). Prison Re-offending Statistics 2019. URL
Central Statistics Office Ireland. (2022b). Probation Re-Offending Statistics 2018. URL

Reporting agencies
Central Statistics Office URL

Population
☑ Released prisoners
☑ Community-sentenced individuals

Reported outcomes
☑ Reconviction
☐ Re-arrest
☐ Reimprisonment
☐ Other (…)

Follow-up periods
☑ 1 year
☑ 2 years
☑ 3 years
☐ Other (…)

Cohort sizes
Released prisoners (in 2019): 4,026
Released prisoners (in 2016): 2,626
Community-sentenced individuals (in 2018): 4,999
Community-sentenced individuals (in 2017): 4.909
Community-sentenced individuals (in 2016): 4.447

Recidivism rates
1-year reconviction rate in released prisoners: 44.6%
3-year reconviction rate in released prisoners: 62.3%
1-year reconviction rate in released prisoners: 28.0%
2-year reconviction rate in released prisoners: 41.0%
3-year reconviction rate in released prisoners: 48.0%

Outcome definition
- Re-conviction: those who re-offend after receiving a sentence. Additional period for 1-2 years added after the main follow-up period for the finalisation of a sentence (Central Statistics Office Ireland, 2022a, 2022b).

Factors contributing to the recidivism rate estimates
- The most recent estimates for the outcome and reporting year are used.

References

Central Statistics Office Ireland. (2018). *Population and Migration Estimates*. https://www.cso.ie/en/releasesandpublications/er/pme/populationandmigrationestimatesapril2018/
Central Statistics Office Ireland. (2022a). *Prison Re-offending Statistics 2019*. https://www.cso.ie/en/releasesandpublications/ep/p-pros/prisonre-offendingstatistics2019/detailsof1-yearcustodialre-offending/
Central Statistics Office Ireland. (2022b). *Probation Re-Offending Statistics 2018*. Retrieved 10.05.2022 from https://www.cso.ie/en/releasesandpublications/ep/p-prs/probationre-offendingstatistics2018/
World Prison Brief. (2023). *Ireland*. Retrieved 04/05/2023 from https://www.prisonstudies.org/country/Ireland-republic

Israel

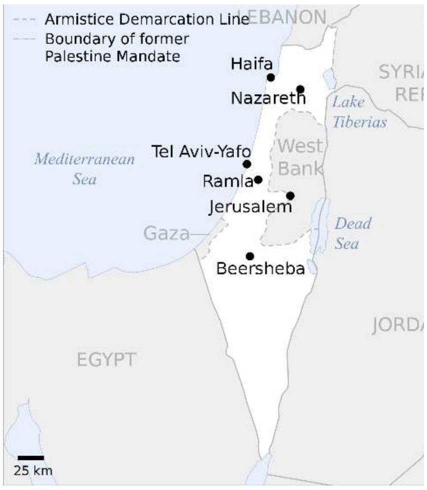

Reporting period
2014-2019

Relevant statistics for the country (in 2016)
Population: 8.502 million (Central Bureau of Statistics, 2016)
Average prison population: 20,368 (World Prison Brief, 2022)
Imprisonment rate: 253 per 100,000 (World Prison Brief, 2022)

Sources for criminal recidivism data
Vaknin, Y, & Ben-Zvi, C. (2021). Recidivism of Israeli Prisoners 2014-2019 URL

Reporting agencies
Israel Prison Service URL

Population
☑ Released prisoners
☐ Community-sentenced individuals

Reported outcomes
☐ Reconviction
☐ Re-arrest
☑ Reimprisonment
☐ Other (…)

Follow-up periods
☑ 1 year
☑ 2 years
☑ 3 years
☑ Other (4 years, 5 years)

Cohort sizes
Released prisoners (in 2019): 6,329
Released prisoners (in 2017): 6,075
Released prisoners (in 2016): 6,549
Released prisoners (in 2015): 6,766
Released prisoners (in 2014): 6,897

Recidivism rates
1-year reimprisonment in released prisoners (2019 cohort): 12.7%
2-year reimprisonment in released prisoners: (2017): 22.5%
3-year reimprisonment in released prisoners: (2016): 27.9%
4-year reimprisonment in released prisoners: (2015): 34.3%
5-year reimprisonment in released prisoners: (2014): 39.2%

Outcome definition
- Reimprisonment: new custodial sentence following the initial release (Vaknin & Ben-Zvi, 2021).

Factors contributing to the recidivism rate estimates
- The most recent estimates for the outcome and reporting year are used.

References

Central Bureau of Statistics. (2016). *Israel Population On The Eve Of 68Th Independence Day - 2016.* https://www.cbs.gov.il/en/mediarelease/pages/2016/israel-population-on-the-eve-of-68th-independence-day-2016.aspx
Vaknin, Y., & Ben-Zvi, K. (2021). *Recidivism of Israeli Prisoners 2014-2019*. https://www.gov.il/BlobFolder/dynamiccollectorresultitem/retsidivisem_2014-2019/he/%2008062021%20-%202019-2014%20פליליים%20אסירים%20של%20רצידיביזם%20דוח(1).pdf
World Prison Brief. (2022). *Israel*. Retrieved 04/06/2023 from https://www.prisonstudies.org/country/israel

Jamaica

Reporting period
2014-2016

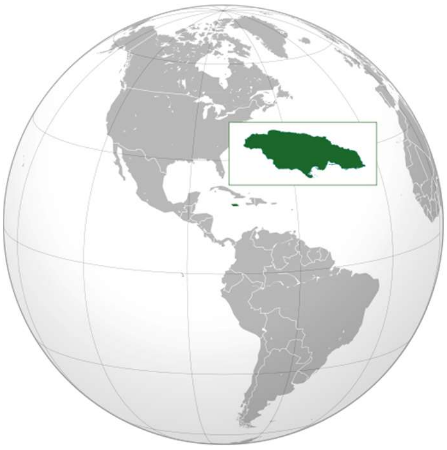

Relevant statistics for the country (in 2015)
Population: 2,727,329 (Statistical Institute of Jamaica, 2018)
Average prison population: 3,860 (World Prison Brief, 2022)
Imprisonment rate: 138 per 100,000 (World Prison Brief, 2022)

Sources for criminal recidivism data
Department of Correctional Services (2017). Improving prisons and reducing recidivism armed with data and information. URL

Reporting agencies
Department of Correctional Services, Jamaica URL

Population
☑ Released prisoners
☐ Community-sentenced individuals

Reported outcomes
☐ Reconviction
☐ Re-arrest
☑ Reimprisonment
☐ Reoffending

Follow-up periods
☐ 1 year
☑ 2 years
☐ 3 years
☐ 5 years

Cohort sizes
Released prisoners: NA

Recidivism rates
2-year reimprisonment rate: 30%

Outcome definition
- Reimprisonment: new prison sentence after initial release (Department of Correctional Services, 2017).

References
Department of Correctional Services. (2017). *Improving prisons and reducing recidivism armed with data and information.* https://www.dcs.gov.jm/pages/recidivism/
Statistical Institute of Jamaica. (2018). *End of Year Population by Parish*. https://statinja.gov.jm/Demo_SocialStats/EndofYearPopulationbyParish.aspx
World Prison Brief. (2022). *Jamaica*. Retrieved 04/05/2023 from https://www.prisonstudies.org/country/jamaica

Japan

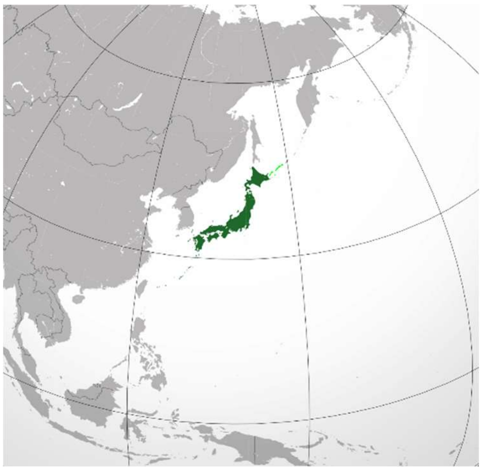

Reporting period
2017

Relevant statistics for the country (in 2018)
Population: 126,706,000 (Statistics Bureau of Japan, 2017)
Average prison population: 50,578 (World Prison Brief, 2021)
Imprisonment rate: 40 per 100,000 (World Prison Brief, 2021)

Sources for criminal recidivism data
Morgan, I., and Morgan, N. (2019). Conference report 2019. URL

Reporting agencies
Asian and Pacific Conference of Correctional URL

Population
☑ Released prisoners
☐ Community-sentenced individuals

Reported outcomes
☐ Reconviction
☐ Re-arrest
☑ Reimprisonment
☐ Reoffending

Follow-up periods
☐ 1 year
☐ 2 years
☑ 3 years
☐ 5 years

Cohort sizes NA

Recidivism rates
3-year reimprisonment rate: 16.9%

Outcome definition

- Reimprisonment: new prison sentence after initial release (Morgan & Morgan, 2019).

Reference

Morgan, I., & Morgan, N. (2019). Conference Report. 39th Asian and Pacific Conference of Correctional Administrators,

Statistics Bureau of Japan. (2017). *Current Population Estimates as of October 1, 2017*. https://www.stat.go.jp/english/data/jinsui/2017np/index.html

World Prison Brief. (2021). *Japan*. Retrieved 04/05/2023 from https://www.prisonstudies.org/country/japan

Korea, Republic of

Reporting period
2017

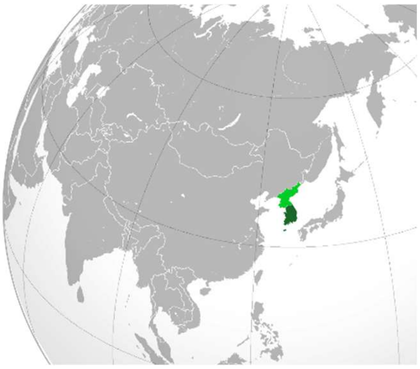

Relevant statistics for the country (in 2016)
Population: 51,217,803 (The World Bank, 2021)
Average prison population: 56,495 (World Prison Brief, 2022)
Imprisonment rate: 112 per 100,000 (World Prison Brief, 2022)

Sources for criminal recidivism data
Recidivism Rate (2022). K-indicator. URL

Reporting agencies
Korean government. URL

Population
☑ Released prisoners
☐ Community-sentenced individuals

Reported outcomes
☐ Reconviction
☐ Re-arrest
☑ Reimprisonment
☐ Other (…)

Follow-up periods
☐ 1 year
☐ 2 years
☑ 3 years
☐ Other (…)

Cohort sizes
Released prisoners: 30,702

Recidivism rates
3-year reconviction in released prisoners: 24.6%

Outcome definition
- Reimprisonment: new prison sentence after initial release (Korean Government, 2022).

References
Korean Government. (2022). *K-indicator: Recidivism Rate*. https://www.index.go.kr/unify/idx-info.do?idxCd=4267
The World Bank. (2021). *Population, total - Korea, Rep.* https://data.worldbank.org/indicator/SP.POP.TOTL?locations=KR
World Prison Brief. (2022). *South Korea*. Retrieved 04/05/2023 from https://www.prisonstudies.org/country/republic-south-korea

Malaysia

Reporting period
2017

Relevant statistics for the country (in 2017-2018)
Population: 32,022,600 (Department of Statistics Malaysia, 2019)
Average prison population: 59,278 (World Prison Brief, 2023)
Imprisonment rate: 188 per 100,000 (World Prison Brief, 2023)

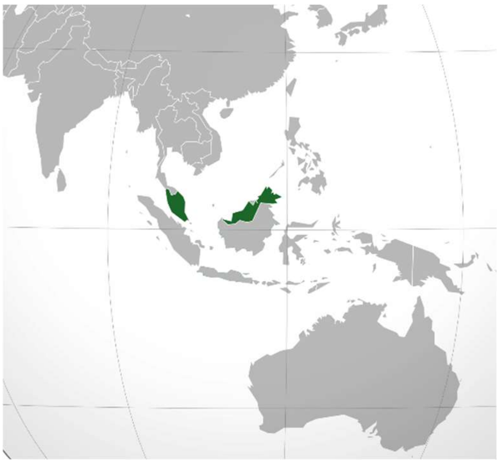

Sources for criminal recidivism data
Wahab, S.Ab. (2019). An introduction to treatment and rehabilitation for illicit drug use offenders in Malaysian prisons. URL

Reporting agencies
Thailand Institute of Justice URL

Population
☑ Released prisoners
☐ Community-sentenced individuals

Reported outcomes
☐ Reconviction
☐ Re-arrest
☑ Reimprisonment
☐ Reoffending

Follow-up periods
☐ 1 year
☐ 2 years
☑ 3 years
☐ 5 years

Cohort sizes
Released prisoners: NA

Recidivism rates
3-year reimprisonment rate: 9.03%

Outcome definition

- Reimprisonment: new prison sentence after initial release (Wahab, 2019)

References

Department of Statistics Malaysia. (2019). *Current Population Estimates, Malaysia, 2018-2019*. https://www.dosm.gov.my/portal-main/release-content/current-population-estimates-malaysia-2018-2019
Wahab, S. A. (2019). *An introduction to treatment and rehabilitation for illicit drug use offenders in Malaysian prisons.* 170th International Training Course of UNAFEI, https://www.unafei.or.jp/publications/pdf/RS_No107/No107_9_IP_Malaysia_1.pdf
World Prison Brief. (2023). *Malaysia*. Retrieved 04/05/2023 from https://www.prisonstudies.org/country/malaysia

Netherlands, the Kingdom of

Reporting period
2017

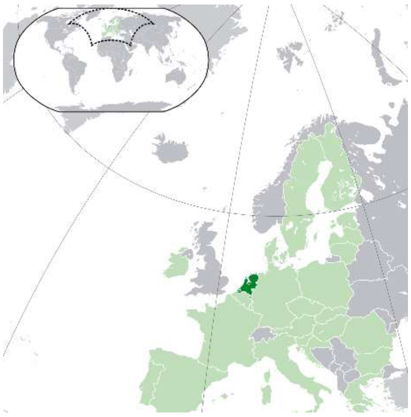

Relevant statistics for the country (in 2018)
Population: 17,080,000 (Statistics Netherlands, 2023)
Average prison population: 10,877 (World Prison Brief, 2021)
Imprisonment rate: 63 per 100,000 (World Prison Brief, 2021)
Gini index: 28.5 (World Bank, 2020)
Homicide rate: 0.8 per 100,000 (Statistics Netherlands, 2020)
Robbery rate: 47 per 100,000 (The Global Economy, 2023)
GDP per capita: 48,675 (World Bank, 2023)

Sources for criminal recidivism data
Ministry of Justice. (2017). Repris URL

Reporting agencies
Ministry of Justice Scientific Research and Documentation Center (WODC)
URL

Population	Reported outcomes	Follow-up periods
☑ Released prisoners	☑ Reconviction	☑ 1 year
☑ Community-sentenced individuals	☐ Re-arrest	☑ 2 years
	☐ Reimprisonment	☐ 3 years
	☐ Other (…)	☐ Other (…)

Cohort sizes
Released prisoners: 23,302
Individuals given community sentences: 36,095

Recidivism rates
1-year reconviction in released prisoners: 18.8%
2-year reconviction in released prisoners: 24.6%
1-year reconviction in community-sentenced individuals: 38.8%
2-year reconviction in community-sentenced individuals: 30.3%

Outcome definition

- Reconviction: any new sentence after the initial release from prison (Ministry of Justice, 2017).

References

Ministry of Justice. (2017). *Repris*. https://wodc-repris.nl/Repris.html
Statistics Netherlands. (2020). *Number of homicides halved over two decades*. https://www.cbs.nl/en-gb/news/2020/42/number-of-homicides-halved-over-two-decades
Statistics Netherlands. (2023). *Population counter*. https://www.cbs.nl/en-gb/visualisations/dashboard-population/population-counter
The Global Economy. (2023). *Netherlands: Robbery rate*. https://www.theglobaleconomy.com/Netherlands/robery/
World Bank. (2020). *Gini index - Netherlands*. https://data.worldbank.org/indicator/SI.POV.GINI?locations=NL
World Bank. (2023). *GDP per capita (current US$) - Netherlands*. https://data.worldbank.org/indicator/NY.GDP.PCAP.CD?locations=NL
World Prison Brief. (2021). *The Netherlands*. Retrieved 04/05/2023 from https://www.prisonstudies.org/country/netherlands

New Zealand

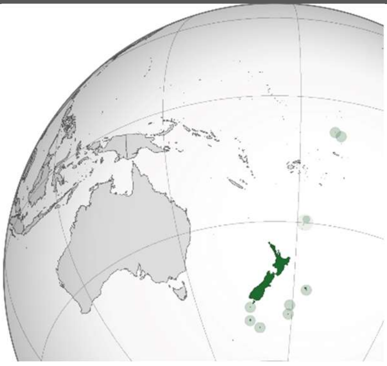

Reporting period
2020-2021

Relevant statistics for the country (in 2020)
Population: 5,025,000 (Stats NZ, 2020)
Average prison population: 9,469 (World Prison Brief, 2023)
Imprisonment rate: 186 per 100,000 (World Prison Brief, 2023)

Sources for criminal recidivism data
New Zealand Department of Corrections (2022). Annual Report. URL

Reporting agencies
New Zealand Department of Corrections URL

Population	Reported outcomes	Follow-up periods
☑ Released prisoners	☑ Reconviction	☑ 1 year
☑ Community-sentenced individuals	☐ Re-arrest	☐ 2 years
	☑ Reimprisonment	☐ 3 years
	☐ Other (…)	☐ 5 years

Cohort sizes
Released prisoners: NA
Individuals given community sentences: NA

Recidivism rates
1-year reimprisonment in released prisoners: 22%
1-year reconviction in released prisoners: 35.8%
1-year reimprisonment in those beginning community sentences: 4.9%
1-year reconviction in those beginning community sentences: 18.9%

Outcome definition
- Reimprisonment: receiving a prison sentence after the being released from prison or since starting a community-based sentence (Department of Corrections, 2022).
- Reconviction: receiving a new sentence after being released from prison or since starting a community-based sentence (Department of Corrections, 2022).

Notes on recidivism rates trends
- Recidivism rates differ from the previous systematic review's report from 2017/2018 where 1-year re-imprisonment in released prisoners was 32.2%, 1-year reconviction in released prisoners was 46.8%, 1-year imprisonment in those beginning community sentences was 5.7%, 1-year reconviction in those beginning community sentences was 27.5% (Department of Corrections, 2018). However, these figures are not comparable to the most recent report as the reporting method changed as of 2020. For the reporting period of 2019-2020, 1-year re-imprisonment in released prisoners was 24%, 1-year reconviction in released prisoners was 38.8%, 1-year imprisonment in those beginning community sentences was 5.9%, 1-year reconviction in those beginning community sentences was 21.2% (Department of Corrections, 2022).

References

Department of Corrections. (2018). *Annual Report*. https://www.corrections.govt.nz/__data/assets/pdf_file/0005/33809/Department_of_Corrections_Annual_Report_2017_2018.pdf

Department of Corrections. (2022). *Annual Report*. https://www.corrections.govt.nz/__data/assets/pdf_file/0010/44398/Annual_Report_2020_2021_Final_Web.pdf

Stats NZ. (2020). *National population estimates: At 30 June 2020*. https://www.stats.govt.nz/information-releases/national-population-estimates-at-30-june-2020

World Prison Brief. (2023). *New Zealand,*. Retrieved 04/05/2023 from https://www.prisonstudies.org/country/new-zealand

Norway

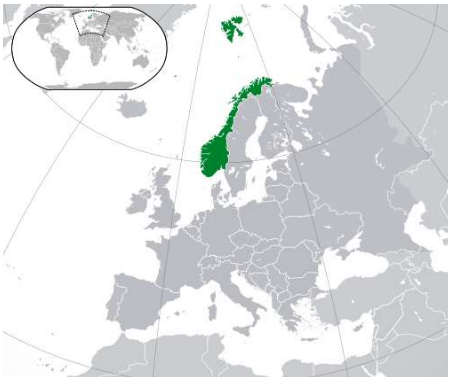

Reporting period
2018

Relevant statistics for the country (in 2018)
Population: 5,296,000 (Statistics Norway, 2018)
Average prison population: 3,425 (World Prison Brief, 2023)
Imprisonment rate: 65 per 100,000 (World Prison Brief, 2023)
Gini index: 27.6 (World Bank, 2020)
Homicide rate: 1.2 per 100,000 (Statistics Norway, 2023)
Robbery rate: 58 per 100,000 (Statistics Norway, 2023)
GDP per capita: 82,268 (World Bank, 2023)

Sources for criminal recidivism data
Kristoffersen, R. (2022). Correctional Statistics of Denmark, Finland, Iceland, Norway and Sweden 2016 – 2020. URL

Reporting agencies
University College of Norwegian Correctional Service URL

Population
☑ Released prisoners
☐ Community-sentenced individuals

Reported outcomes
☑ Reconviction
☐ Re-arrest
☐ Reimprisonment
☐ Other (…)

Follow-up periods
☐ 1 year
☑ 2 years
☐ 3 years
☐ Other (…)

Cohort sizes
Released prisoners: 4,509

Recidivism rates
2-year reconviction in released prisoners: 17.6%

Outcome definition
- Re-conviction: new unconditional sentence within the follow-up period (Kristoffersen, 2022)

References

Kristoffersen, R. (2022). *Correctional Statistics of Denmark, Finland, Iceland, Norway and Sweden 2016 – 2020.* https://www.fangelsi.is/media/almennt/Nordic-Statistics-2016_2020_final.pdf
Statistics Norway. (2018). *This is Norway 2018*. https://www.ssb.no/en/befolkning/artikler-og-publikasjoner/_attachment/364602?_ts=1664418b978
Statistics Norway. (2023). Offences and victims reported to the police. https://www.ssb.no/en/sosiale-forhold-og-kriminalitet/kriminalitet-og-rettsvesen/statistikk/anmeldte-lovbrudd-og-ofre
World Bank. (2020). *Gini index - Norway*. https://data.worldbank.org/indicator/SI.POV.GINI?locations=NO
World Bank. (2023). *GDP per capita (current US$) - Norway*. https://data.worldbank.org/indicator/NY.GDP.PCAP.CD?locations=NO
World Prison Brief. (2023). *Norway*. Retrieved 04/05/2023 from https://www.prisonstudies.org/country/Norway

The Philippines

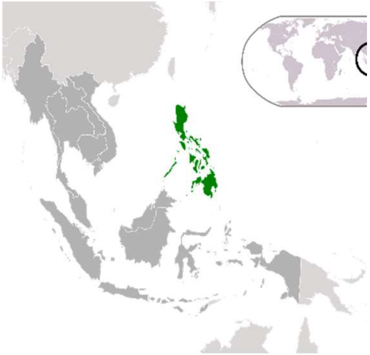

Reporting period
2020

Relevant statistics for the country
Population (in 2020): 112,190,977 (Mactrotrends, 2023)
Average prison population (in 2021): 165,583 (World Prison Brief, 2022)
Imprisonment rate (in 2021): 151 per 100,000 (World Prison Brief, 2022)

Sources for criminal recidivism data
Data requested directly from Bureau of Criminal Justice and Penology

Reporting agencies
Bureau of Criminal Justice and Penology [URL]

Population	Reported outcomes	Follow-up periods
☑ Released prisoners	☐ Reconviction	☐ 1 year
☐ Community-sentenced individuals	☐ Re-arrest	☐ 2 years
	☑ Reimprisonment	☐ 3 years
	☐ Other (…)	☑ Other (21 months)

Cohort sizes
Released prisoners: 84,133

Recidivism rates
21 months reimprisonment in released prisoners: 17.5%

Outcome definition
- Reimprisonment: receiving a new prison sentence after being released from prison.

Recidivism

Mactrotrends. (2023). *Philippines Population 1950-2023*. https://www.macrotrends.net/countries/PHL/philippines/population

World Prison Brief. (2022). *The Philippines*. Retrieved 04/05/2023 from https://www.prisonstudies.org/country/philippines

Poland

Reporting period
2012

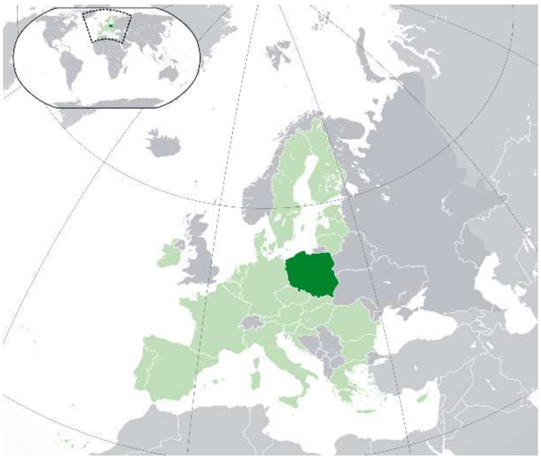

Relevant statistics for the country (in 2012)
Population: 38,533,299 (Statistics Poland, 2012)
Average prison population: 84,156 (World Prison Brief, 2023)
Imprisonment rate: 221 per 100,000 (World Prison Brief, 2023)
Gini index: 33.5 (World Bank, 2020)
Homicide rate: 1.1 per 100,000 (United Nations Office on Drugs and Crime, 2013)
Robbery rate: 43 per 100,000 (The Global Economy, 2018)
GDP per capita: 13,011 (World Bank, 2023)

Sources for criminal recidivism data
Ministry of Justice of Poland (2018). N Pan Marek Kuchciński Marszałek Sejmu Rzeczypospolitej Polskiej. URL

Reporting agencies
Ministry of Justice URL

Population
☑ Released prisoners
☐ Community-sentenced individuals

Reported outcomes
☐ Reconviction
☐ Re-arrest
☐ Reimprisonment
☑ Return to crime

Follow-up periods
☑ 1 year
☑ 2 years
☑ 3 years
☑ 4 years
☑ 5 years

Cohort sizes
Released prisoners: 30,899

Recidivism rates
1-year reconviction in released prisoners: 16.6%
2-year reconviction in released prisoners: 28.3%
3-year reconviction in released prisoners: 35.9%
4-year reconviction in released prisoners: 39.9%
5-year reconviction in released prisoners: 40.4%

Outcome definition
- Return to crime: committing another crime after being released from prison (Ministry of Justice of Poland, 2018).

References

Ministry of Justice of Poland. (2018). *Response to Sejm.* https://orka2.sejm.gov.pl/INT8.nsf/klucz/ATTB68JU5/%24FILE/i26703-o1.pdf
Statistics Poland. (2012). *Population. Size and structure of population and vital statistics by territorial division.* https://stat.gov.pl/en/topics/population/population-size-and-structure-of-population-and-vital-statistics-by-territorial-division-as-of-december-31-2012,3,7.html
The Global Economy. (2018). Poland: Robbery rate. https://www.theglobaleconomy.com/Poland/robery/
United Nations Office on Drugs and Crime. (2013). *Global Study on Homicide 2013.* https://www.unodc.org/documents/data-and-analysis/statistics/GSH2013/2014_GLOBAL_HOMICIDE_BOOK_web.pdf
World Bank. (2020). *Gini index - Poland.* https://data.worldbank.org/indicator/SI.POV.GINI?locations=PL
World Bank. (2023). *GDP per capita (current US$) - Poland.* https://data.worldbank.org/indicator/NY.GDP.PCAP.CD?locations=PL
World Prison Brief. (2023). *Poland*. Retrieved 04/05/2023 from https://www.prisonstudies.org/country/poland

Singapore

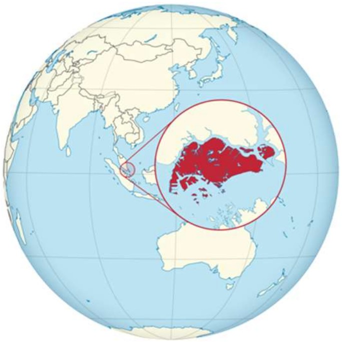

Reporting period
2019

Relevant statistics for the country (in 2018)
Population: 5,640,000 (Statistics Singapore, 2018)
Average prison population: 11,737 (World Prison Brief, 2022)
Imprisonment rate: 199 per 100,000 (World Prison Brief, 2022)
Gini index: 45.2 (World Bank, 2020)
Homicide rate (in 2018): 0.2 per 100,000 (World Population Review, 2023)
Robbery rate: 0.9 per 100,000 (Singapore Police Force, 2020)
GDP per capita: 65,831(World Bank, 2023)

Sources for criminal recidivism data
Singapore Prison Service (2021). SPS Annual Statistics Release for 2021. URL

Reporting agencies
Singapore Prison Service URL

Population
☑ Released prisoners
☐ Community-sentenced individuals

Reported outcomes
☑ Reconviction
☐ Re-arrest
☐ Reimprisonment
☐ Other (…)

Follow-up periods
☐ 1 year
☑ 2 years
☐ 3 years
☐ Other (…)

Cohort sizes
Released prisoners: 10,570

Recidivism rates
2-year reconviction: 37.2%

Outcome definition
- Reconviction: detention, imprisonment, or receiving a day reporting order after being into the custody of the Singapore Prison Service.

References

Singapore Police Force. (2020). Annual Crime Brief 2019 https://www.police.gov.sg/-/media/CDFB635E882E4FBCB85B859FA85845F4.ashx
Statistics Singapore. (2018). *Population Trends 2018*. https://www.singstat.gov.sg/-/media/files/publications/population/population2018.pdf
World Bank. (2020). *Gini index - Singapore*. https://data.worldbank.org/indicator/SI.POV.GINI?locations=SG
World Bank. (2023). *GDP per capita (current US$) - Singapore*. https://data.worldbank.org/indicator/NY.GDP.PCAP.CD?locations=SG
World Population Review. (2023). *Murder Rate by Country*. https://worldpopulationreview.com/country-rankings/murder-rate-by-country
World Prison Brief. (2022). *Singapore*. Retrieved 04/05/2023 from https://www.prisonstudies.org/country/singapore

Sweden

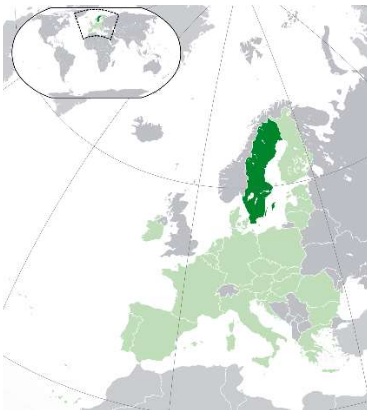

Reporting period
2018-2019

Relevant statistics for the country (in 2018)
Population: 10,230,185 (Statistics Sweden, 2022)
Average prison population: 6,391 (World Prison Brief, 2020)
Imprisonment rate: 63 per 100,000 (World Prison Brief, 2020)
Gini index: 30.0 (World Bank, 2020)
Homicide rate: 1.1 per 100,000 (Swedish National Council for Crime Prevention, 2023)
Robbery rate: 82.7 per 100,000 (Swedish National Council for Crime Prevention, 2023)
GDP per capita: 54,589 (World Bank, 2023)

Sources for criminal recidivism data

Swedish National Council for Crime Prevention. (2019). Återfall i brott Preliminär statistik 2019. URL
Kristoffersen, R. (2022). Correctional Statistics of Denmark, Finland, Iceland, Norway and Sweden 2016 – 2020. URL

Reporting agencies
Swedish National Council for Crime Prevention URL
University College of Norwegian Correctional Service URL

Population
☑ Released prisoners
☐ Community-sentenced individuals

Reported outcomes
☑ Reconviction
☐ Re-arrest
☐ Reimprisonment
☐ Other (…)

Follow-up periods
☑ 1 year
☑ 2 years
☐ 3 years
☐ Other (…)

Cohort sizes
Released prisoners (2018): 7,959
Released prisoners (2019): NA

Recidivism rates
Swedish National Council for Crime Prevention:
1-year reconviction in released prisoners (2019 cohort): 43%
University College of Norwegian Correctional Service:
2-year reconviction in released prisoners (2018 cohort): 32%

Factors contributing to the recidivism rate estimates

- The cohort sizes and rates vary depending on the source of data, University College of Norwegian Correctional Service (Kristoffersen, 2022) vs. (Swedish National Council for Crime Prevention, 2022). This might be due to different data linkage, index sentence operationalisation, and the specific way of data extracted by the end user.

Outcome definition

- Reconviction (1-year). Those who were released from prison who committed a crime that was persecuted within one year of their release.

References
Kristoffersen, R. (2022). *Correctional Statistics of Denmark, Finland, Iceland, Norway and Sweden 2016 – 2020.*

https://www.fangelsi.is/media/almennt/Nordic-Statistics-2016_2020_final.pdf
Statistics Sweden. (2022). *Population and Population Changes 1749–2022*. https://www.scb.se/en/finding-statistics/statistics-by-subject-area/population/population-composition/population-statistics/pong/tables-and-graphs/population-statistics---summary/population-and-population-changes/
Swedish National Council for Crime Prevention. (2022). *2019 Återfall i brott*. https://bra.se/download/18.146acb6517fd55784014b76/1653979808086/Statistikrapport_aterfall_prel_2019.pdf
Swedish National Council for Crime Prevention. (2023). Crime statistics. https://bra.se/bra-in-english/home/crime-and-statistics/crime-statistics.html
World Bank. (2020). *Gini index - Sweden*. https://data.worldbank.org/indicator/SI.POV.GINI?locations=SE
World Bank. (2023). *GDP per capita (current US$) - Sweden*. https://data.worldbank.org/indicator/NY.GDP.PCAP.CD?locations=SE
World Prison Brief. (2020). *Sweden*. Retrieved 04/05/2023 from https://www.prisonstudies.org/country/Sweden

Switzerland

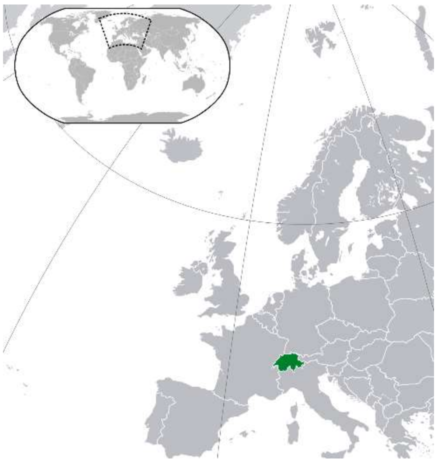

Reporting period
2016

Relevant statistics for the country (in 2016)
Population: 8,372,897 (Population Pyramid, 2022)
Average prison population: 6,912 (World Prison Brief, 2023)
Imprisonment rate: 82 per 100,000 (World Prison Brief, 2023)

Sources for criminal recidivism data
Swiss Federal Statistical Office (2018). Criminal conviction statistics 2018: Recidivism rate. URL

Reporting agencies
Swiss Federal Statistical Office URL

Population
☑ Released prisoners
☐ Community-sentenced individuals

Reported outcomes
☑ Reconviction
☐ Re-arrest
☑ Reimprisonment
☐ Other (…)

Follow-up periods
☐ 1 year
☐ 2 years
☑ 3 years
☐ Other (…)

Cohort sizes
Released prisoners: 1,393

Recidivism rates
3-year reconviction in released prisoners: 44.7%
3-year reimprisonment in released prisoners: 26.1%

Outcome definition
- Reconviction: committing a felony or misdemeanour leading to another conviction within three years of a conviction or release from a custodial sentence (Swiss Federal Statistical Office, 2018).
- Reimprisonment: committing a felony or misdemeanour leading to another conviction and their subsequent imprisonment(Swiss Federal Statistical Office, 2018).

Recidivism

Population Pyramid. (2022). *Switzerland - 2016*. https://www.populationpyramid.net/switzerland/2016/
Swiss Federal Statistical Office. (2018). *Criminal conviction statistics 2018: Recidivism rate.* https://www.bfs.admin.ch/bfs/en/home/statistics/crime-criminal-justice/recidivism.html
World Prison Brief. (2023). *Switzerland*. Retrieved 04/05/2023 from https://www.prisonstudies.org/country/Switzerland

Taiwan

Reporting period
2017

Relevant statistics for the country (in 2016)
Population: 23,594,471 (Mactrotrends, 2023)
Average prison population: 62,398 (World Prison Brief, 2023)
Imprisonment rate: 265 per 100,000 (World Prison Brief, 2023)

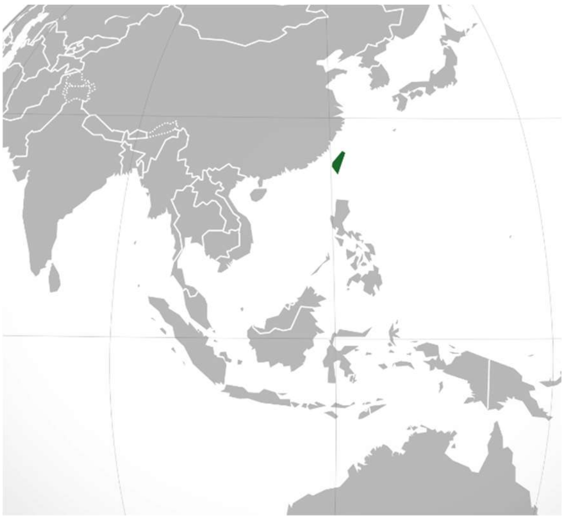

Sources for criminal recidivism data

Tsai, I.-C., & Wu, Y.-T. (2022). Recent Analysis on Crime Trends and Observations on Policy Development in Taiwan. URL

Reporting agencies
Ministry of Justice URL

Population
☑ Released prisoners
☐ Community-sentenced individuals

Reported outcomes
☑ Reconviction
☐ Re-arrest
☐ Reimprisonment
☐ Other (…)

Follow-up periods
☑ 1 year
☑ 2 years
☑ 3 years
☑ Other (6 months, 4 years)

Cohort sizes
Released prisoners: NA

Recidivism rates
6 months reoffending in released prisoners (2018 cohort): 11.1%
1-year reoffending in released prisoners (2017 cohort): 31.0%
2-year reoffending in released prisoners (2016 cohort): 52.0%
3-year reoffending in released prisoners (2015 cohort): 52.4%
4-year reoffending in released prisoners (2014 cohort): 56.6%

Outcome definition

- Reconviction: committing a new crime after being released from prison (Tsai & Wu, 2022).

References

Mactrotrends. (2023). *Taiwan Population 1950-2023*. https://www.macrotrends.net/countries/TWN/taiwan/population

Tsai, I.-C., & Wu, Y.-T. (2022). *Recent Analysis on Crime Trends and Observations on Policy Development in Taiwan*. https://www.tpi.moj.gov.tw/media/208454/1090203-recent-analysis-on-crime-trends-and-observations-on-policy-development-in-taiwan.pdf?mediaDL=true

World Prison Brief. (2023). *Taiwan*. Retrieved 04/05/2023 from https://www.prisonstudies.org/country/taiwan

Thailand

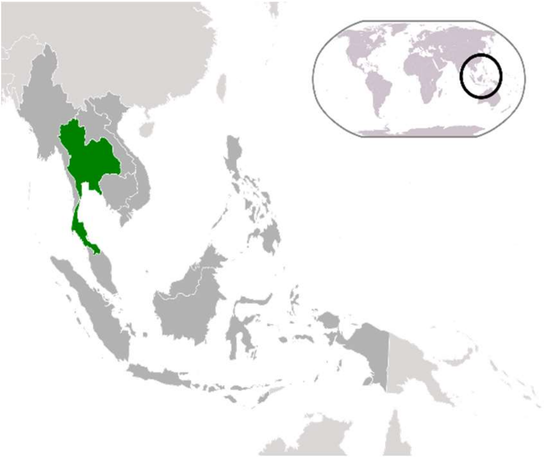

Reporting period
2016

Relevant statistics for the country (in 2016)
Population: 70,697,037 (The World Bank, 2021)
Average prison population: 300,868 (World Prison Brief, 2023)
Imprisonment rate: 437 per 100,000 (World Prison Brief, 2023)

Sources for criminal recidivism data
Thailand Institute of Justice, UNODC (2021). Research on the Causes of Recidivism in Thailand URL

Reporting agencies
Thailand Institute of Justice URL
UNODC URL

Population
☑ Released prisoners
☐ Community-sentenced individuals

Reported outcomes
☐ Reconviction
☐ Re-arrest
☑ Reimprisonment
☐ Other (…)

Follow-up periods
☐ 1 year
☐ 2 years
☑ 3 years
☐ Other (…)

Cohort sizes
Released prisoners: NA

Recidivism rates
3-year reimprisonment in released prisoners: 35.4%

Outcome definition
- Recidivism (3-years). Return to prison after being released from prison (Thailand Institute of Justice, 2021).

Recidivism

Thailand Institute of Justice, U. (2021). *Research on the Causes of Recidivism in Thailand*. https://knowledge.tijthailand.org/en/publication/detail/research-on-the-causes-of-recidivism-in-thailand#book/

The World Bank. (2021). *Population, total - Thailand*. https://data.worldbank.org/indicator/SP.POP.TOTL?locations=TH

World Prison Brief. (2023). *Thailand*. Retrieved 04/05/2023 from https://www.prisonstudies.org/country/Thailand

Scotland

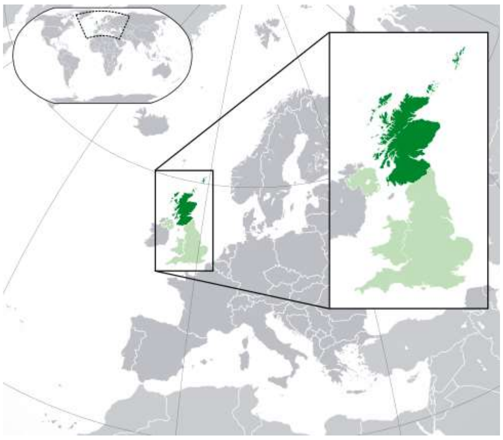

Reporting period
2018-2019

Relevant statistics for the country (in 2018)
Population: 5,438,100 (National Records of Scotland, 2018)
Average prison population: 7,595 (World Prison Brief, 2023)
Imprisonment rate: 140 per 100,000 (World Prison Brief, 2023)

Sources for criminal recidivism data
Scottish Government (2021) Reconviction Rates in Scotland: 2018-19 Offender Cohort URL

Reporting agencies
Scottish Government URL

Population
☑ Released prisoners
☑ Community-sentenced individuals

Reported outcomes
☑ Reconviction
☐ Re-arrest
☐ Reimprisonment
☐ Other (…)

Follow-up periods
☑ 1 year
☐ 2 years
☐ 3 years
☐ Other (…)

Cohort sizes
Released prisoners: 5,549
Community-sentenced individuals: 27,210

Recidivism rates
1-year reconviction in released prisoners: 43.8%
1-year reconviction in community-sentenced individuals: 25.2%

Outcome definition

- Proven reoffending: new conviction after a release from prison of being given a community sentence. Additional 6 months are allowed for finalisation of a sentence (Scottish Government, 2021).

Notes on recidivism rates trends

- In the previous systematic review, the one-year proven reoffending rate for 2015-2016 was reported as 43.7%.

References
National Records of Scotland. (2018). *Scotland's Population: The Registrar General's Annual Review of Demographic Trends*. https://www.nrscotland.gov.uk/files/statistics/rgar/2018/rgar18.pdf
Scottish Government. (2021). *Reconviction Rates in Scotland: 2018-19 Offender Cohort*. https://www.gov.scot/publications/reconviction-rates-scotland-2018-19-offender-cohort/#:~:text=The%20reconviction%20rate%2C%20which%20is,26.4%25%20in%202017%2D18
World Prison Brief. (2023). *United Kingdom: Scotland*. Retrieved 04/05/2023 from https://www.prisonstudies.org/country/united-kingdom-scotland

UK: England and Wales

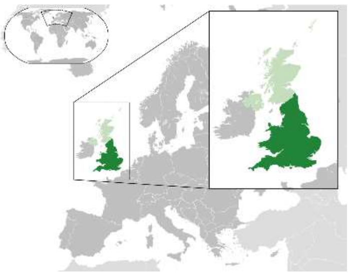

Reporting period
October 2020 – December 2020

Relevant statistics for the country (in 2020)
Population: 59,720,000 (Office for National Statistics, 2020)
Average prison population: 79,514 (World Prison Brief, 2023)
Imprisonment rate: 133 per 100,000 (World Prison Brief, 2023)

Sources for criminal recidivism data
Ministry of Justice (2022). Proven reoffending statistics: October to December 2020. URL

Reporting agencies
Ministry of Justice Website

Population
☑ Released prisoners
☑ Community-sentenced individuals

Reported outcomes
☑ Reconviction
☐ Re-arrest
☐ Reimprisonment
☐ Other (…)

Follow-up periods
☑ 1 year
☐ 2 years
☐ 3 years
☐ Other (…)

Cohort sizes
Released prisoners: 11,820
Community-sentenced individuals: 25,544

Recidivism rates
1-year reconviction in released prisoners: 37.2%
1-year reconviction in community-sentenced individuals: 26.2%

Outcome definition

- Proven reoffending: 'any offence committed in a one-year follow-up period that leads to a court conviction, caution, reprimand or warning in the one-year follow-up or within a further six-month waiting period to allow the offence to be proven in court' (Ministry of Justice, 2022).

Factors contributing to the recidivism rate estimates

- There was a shift toward depenalisation and diversion of drug offenders in the UK (Hamilton, 2022)
- Individuals who committed theft and summary crimes constitute the most of the cohort.

Notes on recidivism rates trends (if available)

- Ministry of Justice notes that the adult reoffending rates remained 'broadly flat' in 2009-2018. The recidivism rates demonstrated volatility attributed by the agency to the effects of Covid-19 pandemic. Two pandemic-related factor were considered as explanations: (1) the higher number of outstanding court cases and (2) the effect of the national lockdown on crime and policing.
- The number of proven drug-related offences were on decline from 2010/2011. This was attributed to the decline in police recorded drug offences (Allen & Tunnicliffe, 2021). However, the number of recorded drug offences started to rise in again 2019/2020, which will likely also influence the upcoming reoffending statistics.

References

Allen, G., & Tunnicliffe, R. (2021). *Drug Crime: Statistics for England and Wales*. https://researchbriefings.files.parliament.uk/documents/CBP-9039/CBP-9039.pdf

Hamilton, I. (2022). The UK tinkers around the edges of drug policy but it still needs national reform. In (Vol. 376): British Medical Journal Publishing Group.

Ministry of Justice. (2022). *Proven reoffending statistics: October to December 2020*. https://www.gov.uk/government/statistics/proven-reoffending-statistics-october-to-december-2020/proven-reoffending-statistics-october-to-september-2020

Office for National Statistics. (2020). *Population estimates for the UK, England and Wales, Scotland and Northern Ireland: mid-2020*. https://www.ons.gov.uk/peoplepopulationandcommunity/populationandmigration/populationestimates/bulletins/annualmidyearpopulationestimates/mid2020

World Prison Brief. (2023). *United Kingdom: England & Wales*. Retrieved 04/05/2023 from https://www.prisonstudies.org/country/united-kingdom-england-wales

UK: Northern Ireland

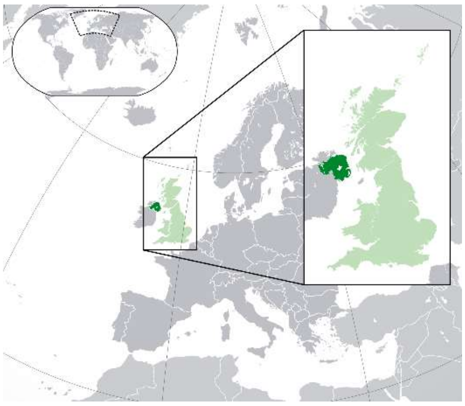

Reporting period
2018-2019

Relevant statistics for the country (in 2018)
Population: 1,881,600 (Northern Ireland Statistical and Research Agency, 2018)
Average prison population: 1,521 (World Prison Brief, 2022)
Imprisonment rate: 81 per 100,000 (World Prison Brief, 2022)

Sources for criminal recidivism data

Department of Justice, Northern Ireland Government (2021) Adult and Youth Reoffending In Northern Ireland 2018-19 cohort URL

Reporting agencies
Department of Justice, Northern Ireland Government URL

Population
☑ Released prisoners
☑ Community-sentenced individuals

Reported outcomes
☑ Reconviction
☐ Re-arrest
☐ Reimprisonment
☐ Other (…)

Follow-up periods
☑ 1 year
☐ 2 years
☐ 3 years
☐ Other (…)

Cohort sizes
Released prisoners: 1,309
Community-sentenced individuals: 3,308

Recidivism rates
1-year reconviction in released prisoners: 44.9%
1-year reconviction in community-sentenced individuals: 21.2%

Outcome definition

- Proven reoffending: new conviction after a release from prison of being given a community sentence. Additional 6 months are allowed for finalisation of a sentence(Department of Justice, 2021).

Notes on recidivism rates trends (if available)

- In the previous systematic review, the one-year proven reoffending rate for 2014-2015 was reported as 37.4%.

References

Department of Justice. (2021). *Adult and Youth Reoffending In Northern Ireland 2018-19 cohort*. https://www.justice-ni.gov.uk/publications/adult-and-youth-reoffending-northern-ireland-201819-cohort

Northern Ireland Statistical and Research Agency. (2018). *2018 Mid-year Population Estimates for Northern Ireland*. https://www.nisra.gov.uk/sites/nisra.gov.uk/files/publications/MYE18-Bulletin.pdf

World Prison Brief. (2022). *United Kingdom: Northern Ireland*. Retrieved 04/05/2023 from https://www.prisonstudies.org/country/united-kingdom-northern-ireland

USA

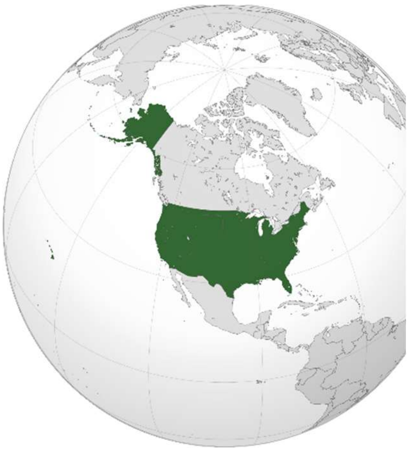

Reporting period
2012

Relevant statistics for the country (in 2012)
Population: 313,998,379 (Country Economy, 2012)
Average prison population: 2,228,424 (World Prison Brief, 2019)
Imprisonment rate: 707 per 100,000 (World Prison Brief, 2019)
Gini index: 40.9 (World Bank, 2020)
Homicide rate: 4.7 per 100,000 (The Global Economy, 2023a)
Robbery rate: 113 per 100,000 (The Global Economy, 2023b)
GDP per capita: 51,784 (World Bank, 2023)

Sources for criminal recidivism data
U.S. Department of Justice. (2021). Recidivism of Prisoners Released in 34 States in 2012: A 5-Year Follow-Up Period. URL

Reporting agencies
U.S. DoJ Bureau of Justice Statistics URL

Population
☑ Released prisoners
☐ Community-sentenced individuals

Reported outcomes
☑ Reconviction
☐ Re-arrest
☐ Reimprisonment
☐ Other (…)

Follow-up periods
☑ 1 year
☑ 2 years
☑ 3 years
☑ Other (4 years, 5 years)

Cohort sizes
Released state prisoners in 34 states: 408,300

Recidivism rates
1-year reconviction in released prisoners: 36.8%
2-year reconviction in released prisoners: 52.9%
3-year reconviction in released prisoners: 61.5%
4-year reconviction in released prisoners: 67.0%
5-year reconviction in released prisoners: 70.8%

Outcome definition

- Reconviction (being convicted of a crime) after release from prison, within 1, 2, 3, 4, or 5 years (U.S. Department of Justice, 2021).

References

Country Economy. (2012). *United States - Population*. https://countryeconomy.com/demography/population/usa?year=2012
The Global Economy. (2023a). *USA: Homicide rate*. https://www.theglobaleconomy.com/USA/homicide_rate/
The Global Economy. (2023b). *USA: Robbery rate*. https://www.theglobaleconomy.com/USA/robery/
U.S. Department of Justice. (2021). *Recidivism of Prisoners Released in 34 States in 2012: A 5-Year Follow-Up Period (2012–2017)*. https://bjs.ojp.gov/sites/g/files/xyckuh236/files/media/document/rpr34s125yfup1217.pdf
World Bank. (2020). *Gini index - USA*. https://data.worldbank.org/indicator/SI.POV.GINI?locations=US
World Bank. (2023). *GDP per capita (current US$) - USA*. https://data.worldbank.org/indicator/NY.GDP.PCAP.CD?locations=US
World Prison Brief. (2019). *USA*. Retrieved 04/05/2023 from https://www.prisonstudies.org/country/united-states-america
